# NFATc1 supports imiquimod-induced skin inflammation by suppressing IL-10 synthesis in B cells

**DOI:** 10.1038/ncomms11724

**Published:** 2016-05-25

**Authors:** Hani Alrefai, Khalid Muhammad, Ronald Rudolf, Duong Anh Thuy Pham, Stefan Klein-Hessling, Amiya K. Patra, Andris Avots, Valesca Bukur, Ugur Sahin, Stefan Tenzer, Matthias Goebeler, Andreas Kerstan, Edgar Serfling

**Affiliations:** 1 Department of Molecular Pathology, Institute of Pathology, Julius-Maximilians-University, Würzburg D-97080, Germany; 2 Department of Dermatology, Venereology and Allergology, University Hospital Würzburg, Würzburg D-97080, Germany; 3 Faculty of Medicine, Department of Medical Biochemistry, Liver Laboratory, Mansoura University, Mansoura 35516, Egypt; 4 TRON gGmbH-Translational Oncology, Johannes-Gutenberg-University Medical Center, Mainz D-55131, Germany; 5 Johannes-Gutenberg-University Medical Center gGmbH, Mainz D-55131, Germany; 6 Institute for Immunology, University Medical Center, Johannes-Gutenberg-University, Mainz D-55131, Germany

## Abstract

Epicutaneous application of Aldara cream containing the TLR7 agonist imiquimod (IMQ) to mice induces skin inflammation that exhibits many aspects of psoriasis, an inflammatory human skin disease. Here we show that mice depleted of B cells or bearing interleukin (IL)-10-deficient B cells show a fulminant inflammation upon IMQ exposure, whereas ablation of NFATc1 in B cells results in a suppression of Aldara-induced inflammation. *In vitro*, IMQ induces the proliferation and IL-10 expression by B cells that is blocked by BCR signals inducing NFATc1. By binding to HDAC1, a transcriptional repressor, and to an intronic site of the *Il10* gene, NFATc1 suppresses IL-10 expression that dampens the production of tumour necrosis factor-α and IL-17 by T cells. These data indicate a close link between NFATc1 and IL-10 expression in B cells and suggest NFATc1 and, in particular, its inducible short isoform, NFATc1/αA, as a potential target to treat human psoriasis.

## Abstract (web summary)

Regulatory B cells are important for preventing skin autoimmunity. Here the authors show that NFATc1 suppresses IL-10 transcription in regulatory B cells, and inhibiting NFATc1 decreases immunopathology in a mouse model of imiquimod-induced skin inflammation.

## Graphical abstract

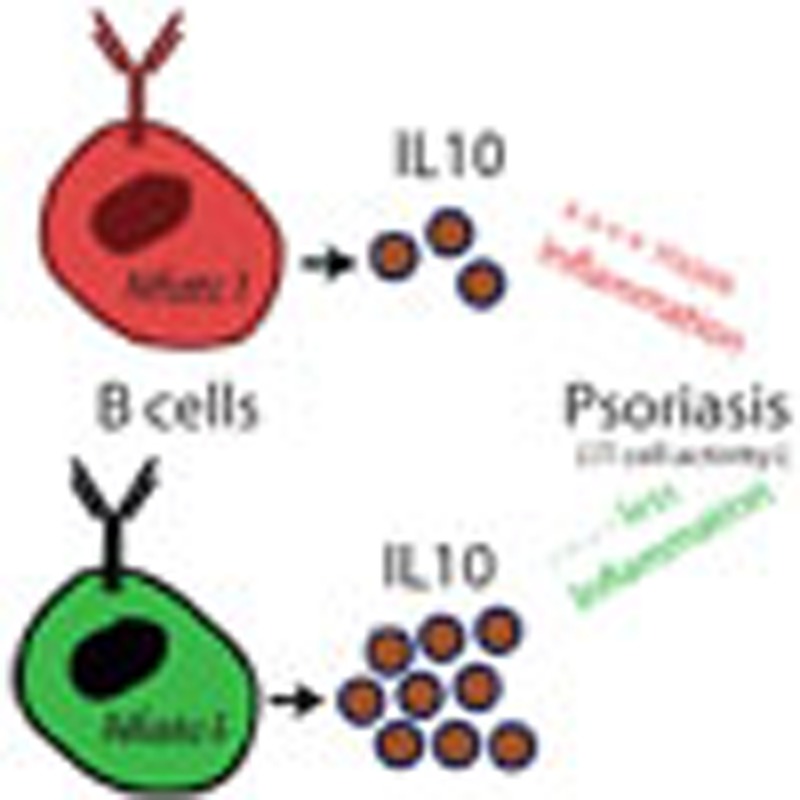

Psoriasis is a chronic inflammatory skin disease that affects 2–3% of the population in Western countries^1^,^2^. It is characterized by the uncontrolled hyperproliferation of keratinocytes (KCs) in the epidermal skin layer that gives rise to erythematous scaly patches. The classical cellular reaction of psoriasis is versatile and involves KCs, dendritic cells (DCs), T lymphocytes, natural killer cells, macrophages and mast cells^2^. Since B cells are hardly detected in psoriatic skin, until recently^3^ their role in psoriasis remained unregarded. It has been assumed that the pathogenesis of psoriasis includes a decrease in tolerance towards self-antigens^4^. A genetic predisposition to injury-induced activation of KCs may trigger psoriasis. Stressed KCs release cytokines (for example, interleukin (IL)-1, IL-6, IL-18 and tumour necrosis factor-α (TNF-α)) and antimicrobial peptides that recruit macrophages and neutrophils to sites of evolving inflammation. Cytokines lead to abnormal KC maturation and activation of DCs^5^,^6^. Plasmacytoid DCs that are known to be involved in antiviral responses have been implicated in the psoriasis reaction. Plasmacytoid DCs contribute to the psoriatic events through endosomal TLR7 and TLR9 signalling. Monocyte-derived mDCs activate different subsets of T cells, most importantly Th1, Th17 and Th22 cells^7^. These activated T-cell subsets release TNF-α, IL-17 and IL-22 that recruit more inflammatory cells and generate an exaggerated state of KC proliferation leading to the clinical picture of psoriatic skin^6^,^8^.

Imiquimod (IMQ) is a potent agonist of TLR7 in mice and TLR7 and TLR8 in humans that has initially been introduced for the treatment of genital warts^9^. Since the development of psoriasis-like skin inflammation was reported as a side effect of IMQ application, IMQ-induced skin inflammation was applied as a mouse model to study human psoriasis^10^. The skin of mice treated with IMQ shows many albeit not all characteristics of psoriatic skin, for example, acanthosis, papillomatosis, inflammatory cell infiltrates and altered dermal vascularity. It is now widely accepted that the topical application of IMQ-containing Aldara cream to the skin of mice is a rapid and cost-effective model for studying early events of psoriasis^11^,^12^.

The immunosuppressant cyclosporin A (CsA) is approved for the treatment of moderate to severe psoriasis^13^. By blocking the activity of the Ser/Thr-specific phosphatase calcineurin (CN), CsA prevents dephosphorylation and, thereby, activation of cytosolic NFAT proteins. Although NFATs are not the only proteins that are dephosphorylated by CN, it is commonly accepted that CN/NFAT complexes are the predominant molecular targets through which CsA blocks the immune system. NFATs represent a family of five transcription factors that share a common DNA-binding domain of approximately 300 amino-acid (aa) residues, the Rel homology (or similarity) domain. In lymphocytes, three out of the four genuine NFATc members, NFATc1, c2 and 3 (which are also known as NFAT2, 1 and 4, respectively), are expressed and controlled by signals emerging from immune receptors. The activation of immune cells via their immune receptors leads to the release of Ca^++^ from intracellular stores, the influx of Ca^++^ through calcium release-activated channels and the rapid activation of CN. Upon complex formation with Ca^++^, calmodulin and further co-factors CN binds to NFAT factors and dephosphorylates their regulatory domain. Thereby, the nuclear localization sequences of NFATs are exposed that drive cytosolic NFAT factors into the nucleus^14^,^15^.

In addition to the rapid nuclear translocation of preformed NFAT factors, immune receptor stimuli also induce the massive generation of NFATc1/αA, a short NFATc1 isoform lacking the C-terminal domain of approximately 250 aa that is common to most other NFAT proteins. NFATc1/αA is the most prominent NFAT protein in nuclei of peripheral T and B lymphocytes activated by immune receptor signals^16^. Because of the ability of NFATc1 to bind to multiple NFAT-binding motifs within a remote intronic enhancer and to composite κB/NFAT sites within the P1 promoter region, the appearance of NFATc1/αA is auto-regulated. This keeps constant high NFATc1/αA levels in lymphocytes during persistent stimulation by immune receptors^16^,^17^,^18^. In contrast to other NFATc proteins that support the induction of anergy and activation-induced cell death of lymphocytes, NFATc1/αA supports the survival of lymphocytes and, thereby, their effector functions^16^,^19^. These and further lines of evidence suggest that in the control of the immune system—including the generation of autoimmune diseases—NFATc1/αA exerts a particular function that differs from that of (most of the) other NFATc factors^19^.

By ablating NFATc1 expression in B cells we show here that NFATc1 supports the development of skin inflammation upon repetitive epicutaneous application of IMQ-containing Aldara cream on the skin of mice. This effect is mediated by IL-10 since mice bearing B cells double-deficient for IL-10 and NFATc1 show no suppression but a fulminant Aldara-mediated skin inflammation. Aldara application to the skin of mice for 1 week leads to an increase in IL-10-producing B10 cells, a decrease in inflammatory cytokines by T cells and to a massive differentiation of splenic B cells to Ab-producing cells. *In vitro*, IMQ treatment of splenic B cells induces IL-10 RNA synthesis within 1–3 days that can be suppressed by the induction of NFATc1 through B cell receptor (BCR) signals. In B cells, NFATc1 binds to the *Il10* gene and dampens, in association with HDAC1, the expression of IL-10 upon IMQ stimulation. These data suggest that targeting NFATc1 induction in B cells might be a novel therapeutic approach to treat psoriasis in humans.

## Results

### Aldara-induced skin inflammation in mice lacking B cells

To elucidate the role of B cells in skin inflammation, we topically applied Aldara, a cream containing 5% IMQ, on the shaved back skin of mice deficient for B cells for 7 consecutive days. Those mice homozygous for *mb1-cre* (*mb1-cre*_*ho*_ mice) bear two mutated *mb1/Cd79a* alleles created by a knock-in of the *cre* gene into the *Cd79a* locus that codes for the BCR α signalling chain. Because of the resulting signalling defect, *mb1-cre*_*ho*_ mice possess a very low number of peripheral B cells (ref. 20 and own observations).

**Figure 1:**
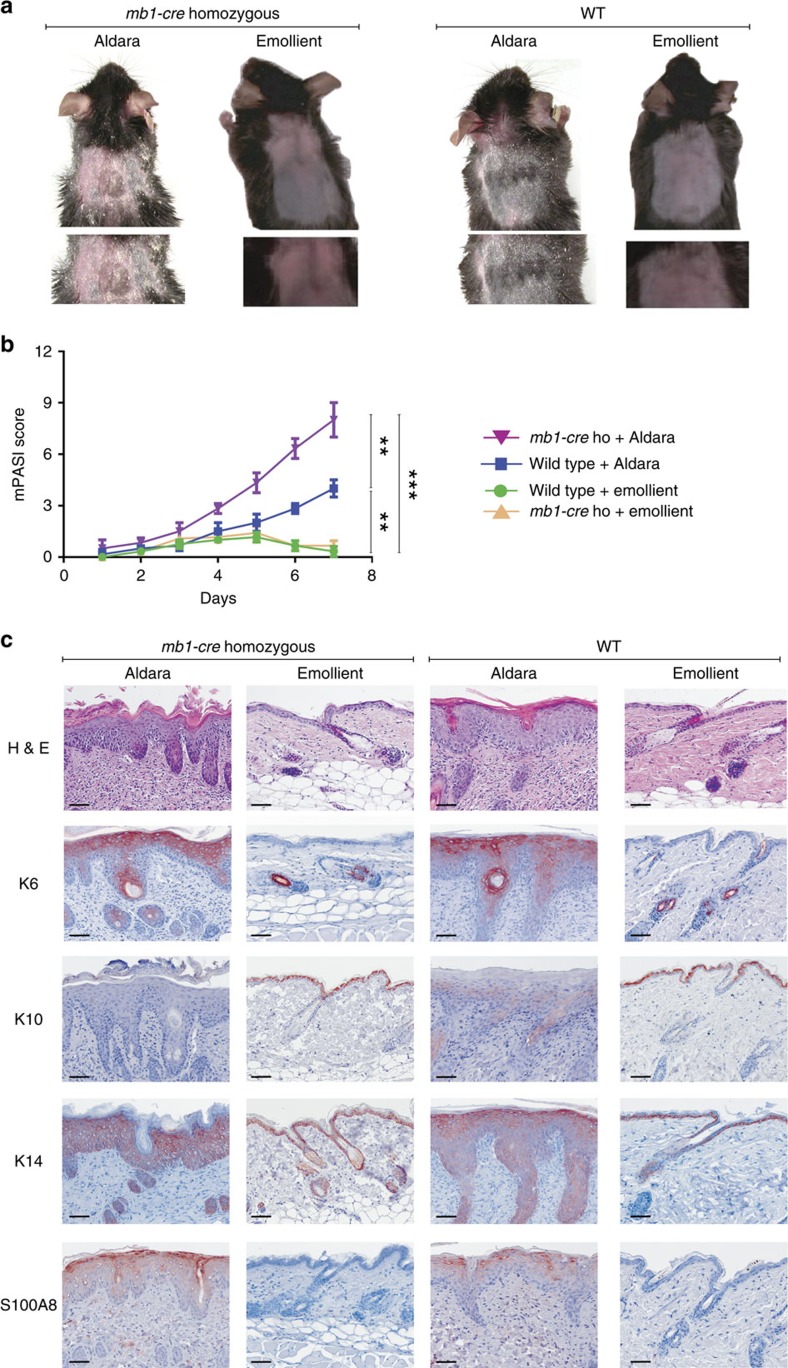
Induction of skin inflammation by Aldara cream in mice lacking B cells. Aldara or emollient cream was applied to the shaved back of mice on 7 consecutive days. *Mb1-cre* homozygous (*mb1-cre* ho) mice possess a minimal number of B cells. Each experiment was done three times with four mice in each group. (**a**) Mice treated with Aldara developed psoriasis-like skin inflammation with erythema, scaling and thickening. (**b**) mPASI reflecting the intensity of skin inflammation. Two-tailed unpaired Student's *t*-test was performed for statistical analysis. Data are shown as means±s.e.m. (**c**) Light microscopy examination of skin sections stained with H&E, or with Abs against the keratins K6, K10 and K14, and S100A8, respectively. Sections of skin treated with Aldara show, in contrast to skin exposed to emollient cream, parakeratosis (retention of nuclei in the ‘stratum corneum'), acanthosis (thickening of the ‘stratum spinosum') and elongation of the dermal papillae. Scale bars, 50 μM. H&E, haematoxylin and eosin.

Aldara application to *mb1-cre*_*ho*_ mice for 7 days induced an exaggerated skin inflammation that is reflected by erythema, scaling and thickening (Fig. 1a). Compared with Aldara-treated wild-type (WT) animals and to mice treated with an emollient cream as control, *mb1-cre*_*ho*_ mice showed a striking increase of skin inflammation as reflected by an increased modified Psoriasis Area and Severity Index (mPASI) adapted to mice (Fig. 1b). Microscopic examination of haematoxylin and eosin-stained skin sections from Aldara-treated *mb1-cre*_*ho*_ mice revealed many histological features of chronic inflammation that are characteristic for human psoriasis including parakeratosis, acanthosis and elongation of the dermal papillae (Fig. 1c). Immunohistochemistry showed an increase in the numbers of KCs expressing keratin K6 and a decrease in K10-expressing cells in the epidermal layer reflecting abnormal differentiation, whereas the enhanced expression of keratin K14 in suprabasal epidermal layers indicated an accelerated KC proliferation. In addition, as compared with WT mice, an increase in the epidermal expression of the inflammatory protein S100A8 was observed in Aldara-treated *mb1-cre*_*ho*_ mice (Fig. 1c). These findings illustrate the importance of B cells in counteracting the development of Aldara-induced inflammatory skin symptoms.

### Attenuated skin inflammation in mice with *Nfatc1* ^*−/−*^ B cells

**Figure 2:**
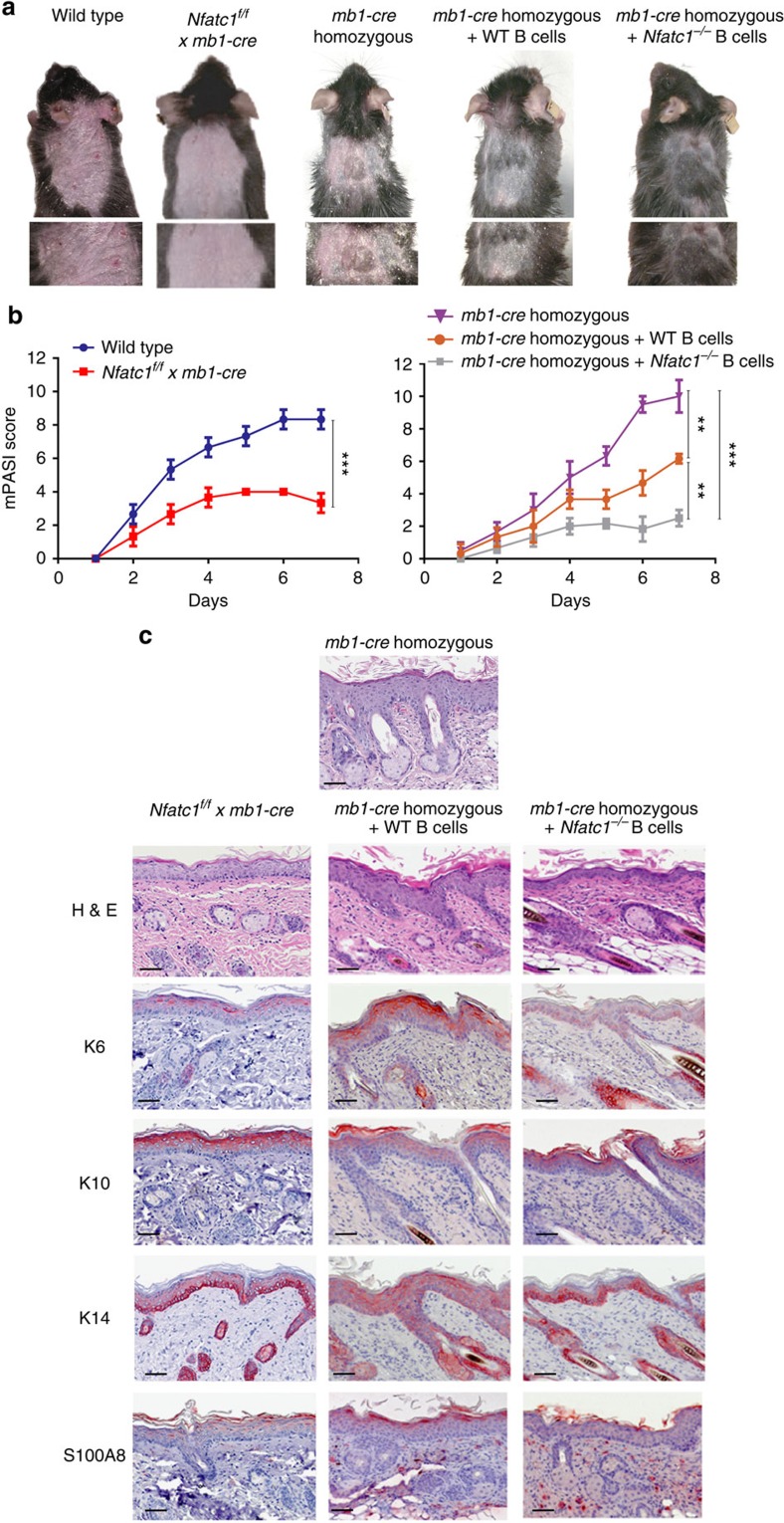
NFATc1 ablation in B cells suppresses the induction of skin inflammation by Aldara. Aldara cream was applied to the shaved backs of mice for 7 days. Splenocytes were isolated from WT mice and mice bearing *Nfatc1*^*−/−*^ B cells and adoptively transferred to *mb1-cre* homozygous mice 3 days before Aldara application. Each experiment was performed three times with four mice in each group. (**a**) Naked eye picture examination of psoriasis-like skin inflammation (erythema, scaling and thickening) in mice treated with Aldara. (**b**) mPASI after application of Aldara. Two-tailed unpaired Student's *t*-test was used for statistical analysis. Data are shown as means±s.e.m. (**c**) Light microscopic examination of skin sections stained with H&E or immunolabeled with Abs directed against K6, K10, K14, or S100A8. Scale bars, 50 μM. H&E, haematoxylin and eosin.

CsA is approved for the systemic treatment of moderate to severe human psoriasis, and co-application of a cream containing 0.1% FK506 (tacrolimus), together with Aldara cream, almost completely suppressed the generation of skin inflammation caused by Aldara (Supplementary Fig. 1). Because NFAT factors are activated through the Ca^++^-CN network that can be blocked efficiently by CsA or FK506, we investigated whether NFATc1, the most prominent NFAT factor in activated B cells, plays a role in Aldara-induced skin inflammation. Upon epicutaneous application of Aldara cream onto *Nfatc1*^*f/f*^ *x mb1-cre* mice bearing NFATc1-deficient B cells^21^, we observed a considerable decrease in inflammation as compared with WT mice (Fig. 2a,b). Upon adoptive transfer of B cells from WT mice into *mb1-cre*_*ho*_ mice 3 days prior Aldara application, we detected an amelioration of the mPASI in those mice that otherwise developed fulminant skin inflammation. Adoptive transfer of *Nfatc1*^*−/−*^ B cells led to a further improvement of the inflammatory symptoms (Fig. 2a,b). These findings are supported by the expression patterns of epidermal K6, K10, K14 and S100A8 in lesional skin (Fig. 2c).

Taken together, these findings illustrate that NFATc1 expression in B cells controls Aldara-induced skin inflammation.

### NFATc1 affects IL-10 production in B cells

**Figure 3:**
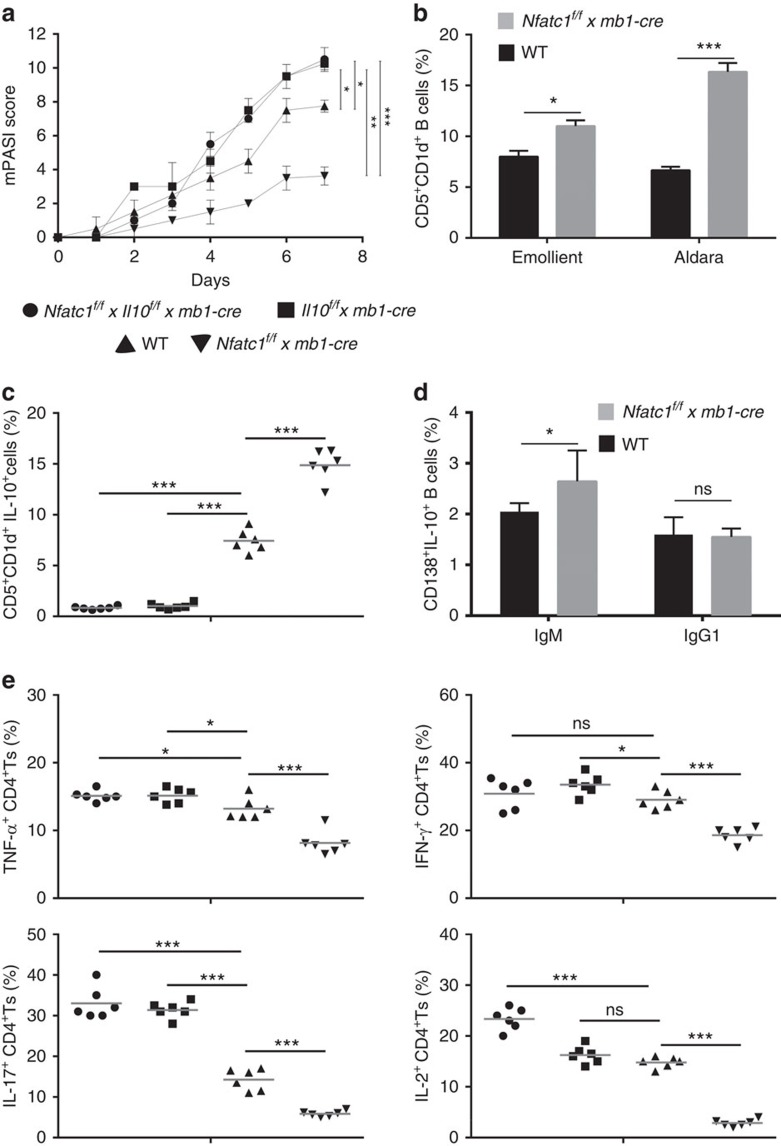
NFATc1 affects the expression of inflammatory cytokines by CD4^+^ T cells via B-cell-derived IL-10. (**a**) Skin inflammation as reflected by mPASI after application of Aldara onto the skin of *Il10*^*f/f*^ *x mb1-cre*, *Il10*^*f/f*^ *x Nfatc1*^*f/f*^ *x mb1-cre*, *Nfatc1*^*f/f*^ *x mb1-cre* and WT mice. (**b**) Increase of CD5^+^CD1d^+^ B cells in WT and *Nfatc1*^*f/f*^ *x mb1-cre* mice upon emollient or Aldara application. (**c**) Increase of splenic B10 cells in mice bearing NFATc1-deficient B cells. Each symbol represents one animal that was treated with Aldara for 7 days. (**d**) Increase of IgM^+^ CD138^+^IL-10^+^ B cells in *Nfatc1*^*f/f*^ *x mb1-cre* mice upon Aldara application for 7 days. (**e**) Decrease in number of CD4^+^T cells expressing inflammatory cytokines in mice bearing NFATc1-deficient B cells. T cells were incubated on α-CD3/CD28 (10 and 3 μg ml^−1^, respectively) for 72 h followed by intracellular staining and flow cytometry. Two-tailed unpaired Student's *t*-test was used for statistical analysis. Data are shown as means±s.e.m.

Several lines of evidence suggested that the suppressive effect of NFATc1 on Aldara-induced skin inflammation might be exerted through IL-10 (ref. 21). To address this, we investigated the effect of Aldara on *Il10*^*f/f*^ *x mb1-cre* mice and on mice bearing B cells double-deficient for IL-10 and NFATc1. Similar to the effect of Aldara on the skin of *mb1-cre*_*ho*_ mice we observed a fulminant skin inflammation in *Il10*^*f/f*^ *x mb1-cre* mice. Moreover, crossing of *Il10*^*f/f*^ *x mb1-cre* mice with *Nfatc1*^*f/f*^ mice indicated that the protective effect of ablated NFATc1 against Aldara-induced inflammation (see Fig. 2a,b) was abrogated in the absence of IL-10 (Fig. 3a and Supplementary Fig. 2). These observations demonstrate that the inflammatory effect of NFATc1 is mediated through the inhibition of IL-10.

CD5^+^CD1d^hi^ B cells have been described as prominent producers of IL-10 (ref. 22). Aldara application to mouse skin led to an increase in the percentage of B cells producing IL-10 (designated as B10 or Breg cells) within the population of splenic B cells^3^. As demonstrated in Fig. 3b,c and in Supplementary Fig. 3A,B, the percentage of B10 cells in spleen, LN and blood of mice bearing *Nfatc1*^*−/−*^ B cells was higher upon Aldara treatment. Another set of IL-10-producing B cells are CD138^+^ plasmablast-like cells consisting mainly of IgM^+^ and IgG1^+^ cells. While the percentage of IgG1^+^ B cells remained constant, the percentage of IgM^+^ plasmablasts increased upon NFATc1 ablation (Fig. 3d).

Effector CD4^+^ T cells are important mediators in the pathogenesis of human psoriasis^23^. To investigate whether the increase in B10 cells in Aldara-treated *Nfatc1*^*f/f*^ *x mb1-cre* mice affects the cytokine production of effector CD4^+^ T cells, we determined the number of CD4^+^ T cells expressing TNFα, IL-17, interferon-γ (IFN-γ) and IL-2 in those mice. To elucidate the IL-10 dependency of cytokine production, we also determined the cytokine levels of mice containing B cells deficient for IL-10, or for both IL-10 and NFATc1. As compared with the WT situation the numbers of TNFα- and IFN-γ-producing CD4^+^ T cells were slightly increased (or remained constant) in mice bearing IL-10- as well as NFATc1/IL-10-deficient B cells (Fig. 3e). In sharp contrast, the proportion of IL-17-secreting CD4^+^ T cells was increased dramatically in those mice as compared with WT mice. Conversely and in line with the moderate inflammatory phenotype, the numbers of CD4^+^ T cells expressing TNFα, IL-17, IFN-γ and IL-2 were largely reduced in *Nfatc1*^*f/f*^ *x mb1-cre* mice when compared with WT as well as to *Il10*^*f/f*^ *x mb1-cre* and *Nfatc1*^*f/f*^ × *Il10*^*f/f*^ x *mb1-cre* mice (Fig. 3e). These data strongly suggest that the production of IL-10 by splenic B cells controls the synthesis of inflammatory cytokines by CD4^+^ T cells.

When we studied the infiltration of lymphocytes and neutrophils into the skin of WT and *Nfatc1*^*f/f*^ x *mb1-cre* mice we observed a reduction in the number of CD3^+^ T cells and Gr-1^+^ neutrophils but an increase in the number of B220^+^ B cells upon Aldara application (Supplementary Fig. 3C,D). From these findings, one may assume that the appearance of B cells affects the mobilization of T cells and neutrophils to inflamed skin lesions.

To investigate the effect of IL-10-producing B cells on T cells by an alternate route, we enriched B10 cells by incubation of splenic B cells with BAFF for 3 days before co-incubation with T cells *in vitro*^24^. Upon incubation of splenic B cells with lipopolysaccaride (LPS) and B-cell activating factor (BAFF) for 3 days *in vitro*, ∼30% of splenic B cells from *Nfatc1*^*f/f*^ *x mb1-cre* mice turned into B10 cells (Supplementary Fig. 4A). In co-culture, such B10 cells reduced the proliferation as well as the production of IFN-γ and, more prominently, TNF-α by CD4^+^ T cells (Supplementary Fig. 4B–D). Upon adoptive transfer of B10 cells into mice, a strong suppressive effect on Aldara-induced skin inflammation was detected, particularly in case of B10 cells derived from *Nfatc1*^*f/f*^ *x mb1-cre* mice (Supplementary Fig. 4E).

These data suggest that (i) high levels of IL-10 produced by B cells suppress Aldara-induced skin inflammation, and (ii) the suppressive effect of NFATc1 ablation on skin inflammation is mediated by an overproduction of IL-10.

### Aldara triggers the differentiation of splenic B cells

**Figure 4:**
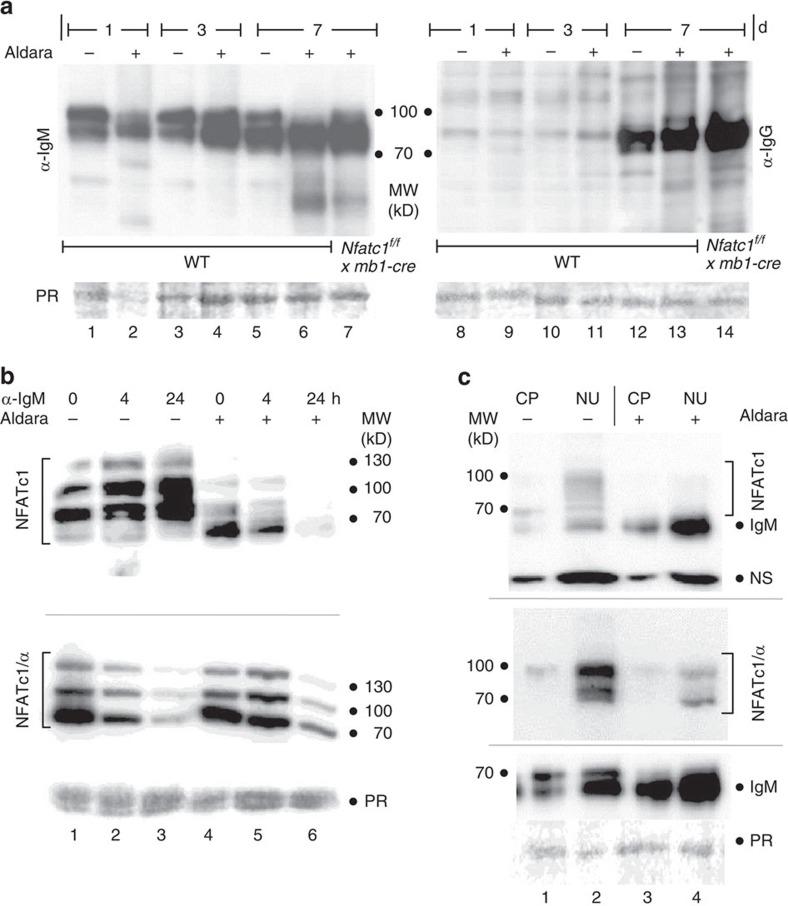
Aldara treatment leads to the differentiation of splenic B cells to Ab-producing cells and affects NFATc1 expression. (**a**) Mice were treated with Aldara (+) or emollient cream (-) for 1, 3 or 7 days. Their splenic B cells were isolated, whole-protein extracts prepared and immunoblotted with Abs raised against murine IgM (lanes 1–7) or IgG (8–14). (**b**) Mice were treated with Aldara (+) or emollient cream (−) for 3 days. Their splenic B cells were left untreated or treated for 4 or 24 h with 10 μg ml^−1^ αIgM mAb in culture. Whole-protein extracts were immunoblotted with Abs raised against NFATc1 (7A6 mAb) (top) or the α-peptide of NFATc1 (bottom). (**c**) Appearance of NFATc1 (uppermost), NFATc1/α (mid) and IgM (below) in cytoplasmic (CP) and nuclear proteins (NU) derived from splenic B cells of mice treated with Aldara (+) or emollient cream (−) for 3 days. PR, ponceau red.

NFATc1 might affect Aldara-mediated IL-10 production of B cells in two ways that are not mutually exclusive: (i) by interfering with the differentiation of splenic B cells to B10 cells; or (ii), more directly, by inhibiting the synthesis of IL-10 in B10 cells. To elucidate whether and how Aldara affects the differentiation of B cells, we immunoblotted protein lysates obtained from splenic B cells of mice treated for 1, 3 or 7 days with Aldara with Abs raised against murine IgM, IgG, IgE or IgA (Fig. 4a and Supplementary Fig. 5). Within 7 days, the splenic B cells of those mice developed from ‘naive' B cells producing only (membranous and secreted) IgM to plasmablast-like cells producing (secreted) IgM, IgG, IgE and IgA. Although Aldara cream exerted the strongest effect on this differentiation program, emollient used as control appeared to stimulate B-cell differentiation as well. This might be in line with a previous report on the TLR7/IMQ-independent, stimulatory activity of Aldara cream on the murine immune system^25^. NFATc1 ablation did not markedly affect the expression of IgM, while it enhanced moderately IgG and IgE production (Fig. 4a and Supplementary Fig. 5). The effect of NFATc1 on IgG was confirmed by incubation of B cells on Kitamura's 3T3 feeder layer cells expressing CD40L and BAFF in the presence of IL-4 (ref. 26). Upon incubation for 4 days, almost twofold more *Nfatc1*^*−/−*^ B cells developed to IgG1^+^ cells than WT cells (Supplementary Fig. 6).

Western blot analysis of NFATc1 expression in splenic B cells obtained from mice treated with emollient for 1–3 days (in Fig. 4b) revealed a certain level of NFATc1/αA, compared with the lack of any NFATc1/αA in (naive) splenic B cells from untreated mice. However, when mice were treated with Aldara for 3–7 days the strong induction of NFATc1/αA by α-IgM for 24 h—as seen in (naive) splenic B cells (Supplementary Fig. 7A)—was abolished (Fig. 4b and Supplementary Fig. 7C). Instead, an increase in IgM was observed (Fig. 4c, bottom). These data find support in B-cell proteins from mice treated for 7 days by Aldara cream that do not show any NFATc1/α but Ig expression instead (see lanes 2, 4 and 6 in Supplementary Fig. 7C). However, when splenic B cells from those mice were treated by TPA+ionomycin instead by α-IgM for 24 h, induction of NFATc1/αA was observed (Supplementary Fig. 7D). This shows that in spite of an overall decrease of NFATc1 levels in splenic B cells from Aldara-treated mice they have the capacity to induce NFATc1/αA.

Taken together, these data indicate that Aldara application for 7 days leads (i) to the differentiation of ‘naive' splenic B cells to plasmablast-like cells that (ii) show a marked alteration in their capacity to induce NFATc1.

### NFATc1 binds to and suppresses the *Il10* gene

**Figure 5:**
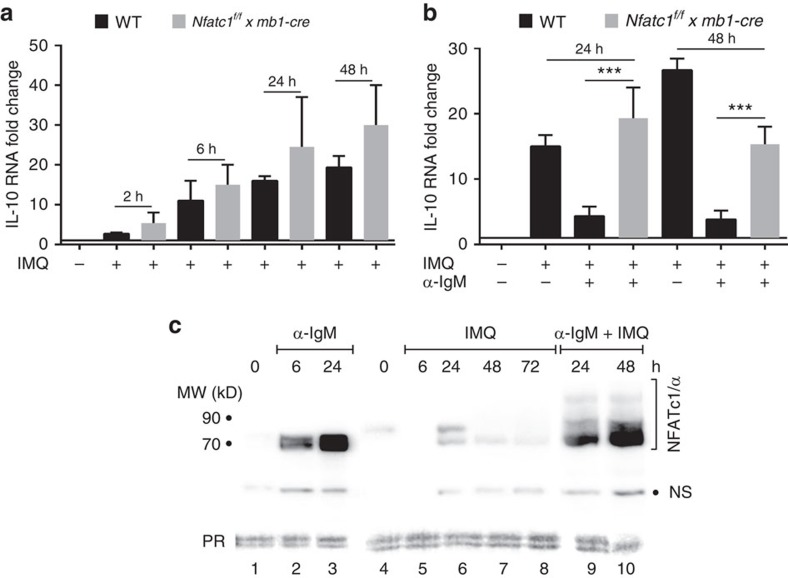
NFATc1 suppresses IL-10 RNA induction in cultured splenic B cells. (**a**) Induction of IL-10 RNA in WT and *Nfatc1*^*−/−*^ splenic B cells by 1 μg ml^−1^ IMQ *in vitro*. Real-time PCR assays. (**b**) Inhibition of IMQ-mediated IL-10 RNA induction upon exposure to αIgM *in vitro*. For A and B, two-tailed unpaired Student's *t*-test was used for statistical analysis. Data are shown as means±s.e.m. (**c**) Immunoblot showing the induction of NFATc1/αA by αIgM-mediated BCR—but not IMQ—signals in B cells *in vitro*. Freshly prepared splenic B cells were left unstimulated (lanes 1 and 4), or stimulated by 10 μg ml^−1^ αIgM for 6 or 24 h (lanes 2 and 3), or by 1 μg ml^−1^ IMQ for 6, 24, 48 and 72 h (lanes 5–8), or by αIgM and IMQ for 24 or 48 h (lanes 9+10), respectively. NS, non-specific band.

Stimulation of splenic B cells by αIgM mAb leads to a rapid increase in IL-10 RNA levels that peak within 2 h but decrease upon further stimulation *in vitro*. A similar, albeit somewhat weaker IL-10 RNA induction was detected in *Nfatc1*^*−/−*^ B cells (Supplementary Fig. 8A). In contrast to αIgM but similar to LPS (Supplementary Fig. 8B), IMQ treatment of freshly prepared splenic B cells resulted in a strong increase in IL-10 RNA levels within 6 and 24 h, which persisted upon stimulation for 48 h (Fig. 5a). This is reflected in the increase of CD5^+^CD1d^+^ B cells among splenic B cells and of approximately twofold more CD5^+^CD1d^+^IL-10^+^ B cells (Supplementary Fig. 8C,D). Since *Nfatc1*^*−/−*^ B cells showed about the same IMQ-mediated induction of IL-10 mRNA as WT B cells, NFATc1 seems to play a minor, if any role in the IMQ-induced synthesis of IL-10 mRNA under these conditions. However, by adding αIgM mAb to IMQ-treated B cell cultures the induction of IL-10 mRNA was strongly suppressed in WT B cells, whereas no (or a weak) suppression was observed in *Nfatc1*^*−/−*^ B cells (Fig. 5b). Since αIgM mAb stimulation of IMQ-treated B cells resulted in a massive induction of NFATc1/αA within 24 h (Fig. 5c, lanes 9+10), these data suggest that depending on the cellular context NFATc1/αA is able to suppress IMQ-mediated induction of IL-10 RNA in B cells.

**Figure 6:**
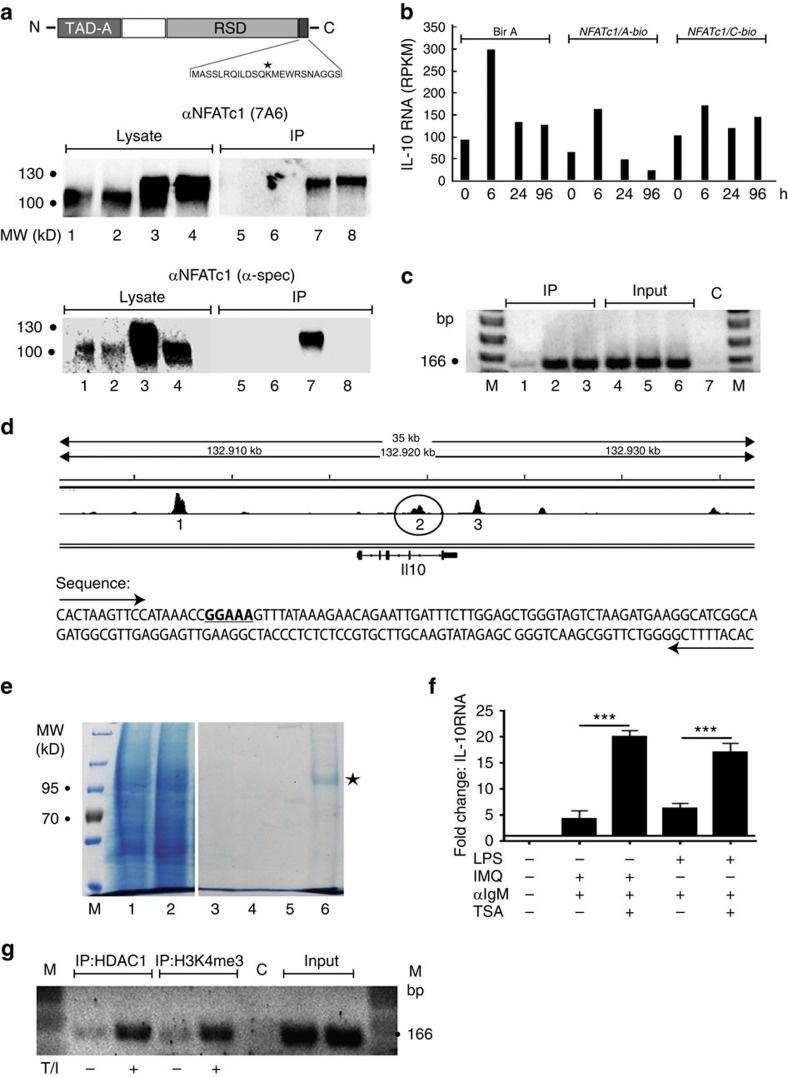
NFATc1 binds to the *Il10* gene. (**a**, top) Scheme of the NFATc1/A-bio-protein expressed in WEHI cells, including the C-terminal bio-tag sequence with lysine that is biotinylated (asterisk). RSD, Rel Similarity Domain; TAD-A, transactivation domain A. (bottom) Expression of NFATc1-bio proteins. Whole proteins (lanes 1–4; lysate) or proteins bound to magnetic streptavidin beads (5–8; IP) were immunoblotted. Lanes 1+5, control cells; lanes 2+6, cells expressing BirA; lanes 3+7, cells expressing BirA+NFATc1/A-bio; lanes 4+8, cells expressing BirA+NFATc1/C+bio. (**b**) NFATc1/αA-bio impairs *Il10* RNA expression in WEHI cells. Cells expressing either the bio-ligase BirA alone (columns 1–4), BirA and NFATc1/A-bio (5–8) or BirA and NFATc1/C-bio (9–12) were left untreated (0) or treated with αIgM for 6, 24 or 96 h. Their RNA was sequenced in NGS assays. RPKM, reads per kilo base per million of IL-10 RNA. (**c**) ChIP assays using chromatin from cells expressing BirA (lanes 1), NFATc1/A-bio+BirA (lane 2) or NFATc1/C-bio+BirA (lane 3). Lanes 4–6, input controls, lane 7, water control. M, nucleotide marker. (**d**) Scheme of the murine *Il10* locus showing the binding sites 1, 2 and 3 for NFATc2/NFAT1 in and around the *Il10* gene in CD8^+^ T cells^27^. Region 2 (encircled) within intron four containing a consensus site for NFAT binding (underlined) was amplified. (**e**) Affinity chromatography for NFATc1/A-bio isolation from WEHI B cells. In lanes 1 and 2, whole protein from cells expressing Bir A (1) or NFATc1/A-bio+BirA (2), in lanes 3+4, protein washes and lanes 5+6, protein bound to beads were fractionated. Asterisk, bound NFATc1/A-bio. NFATc1 partner proteins sequenced in those assays are presented in Supplementary Table 1. (**f**) Splenic B cells were left untreated or treated with αIgM and IMQ or αIgM and LPS for 24 h, followed by co-incubation with 0.5 μM TSA for the last 12 h. Data are shown as means±s.e.m. Two-tailed unpaired *t*-test was used for statistical analysis. (**g**) ChIP assay for the appearance of HDAC1 and the histone mark H3K4me3 at site 2 of *Il10* gene. Splenic B cells were treated with TPA/ionomycin (T/I) for 4 h, and processed as described in Methods section. NGS, next-generation sequencing, TSA, trichostatin A.

To analyse if the transcription factor NFATc1 acts as a direct negative regulator of the *Il10* gene, we used WEHI 231 B lymphoma cells that (over-) express chimaeric NFATc1/A-bio-proteins (Fig. 6a). WEHI 231 cells produce constitutively 100-fold more IL-10 RNA than non-induced primary B cells (Fig. 6b). By co-expressing the biotin-ligase BirA and chimaeric NFATc1-bio proteins, this allows the rapid isolation of crosslinked NFATc1/chromatin complexes in chromatin immunoprecipitation (ChIP) assays (Fig. 6c), and of NFATc1 partners in mass spectrometry (MS) assays using magnetic streptavidin beads (Fig. 6e and Supplementary Table 1). Next-generation sequencing of the transcriptome of αIgM-stimulated WEHI cells showed that ectopic (over-) expression of NFATc1/αA-bio led to a 2–3-fold reduction in *Il10* RNA levels, whereas IL-10 expression in NFATc1/ßC-bio-overexpressing cells remained almost unaffected (Fig. 6b). The negative effect of NFATc1/αA on IL-10 mRNA expression was even more pronounced after α-IgM mAb stimulation, especially after 24 and 96 h as compared with control (BirA) or NFATc1/ßC overexpression.

For the detection of NFATc1 binding to the *Il10* gene, we selected primer pairs of three regions within and around the murine *Il10* gene that have been shown in ChIP seq assays to be bound by NFATc2 in CD8^+^ T cells (Fig. 6d)^27^. Whereas in our individual ChIP assays the primers of regions 1 and 3 located upstream or downstream from the *Il10* gene did not give rise to any PCR product, primers from the last *Il10* intron amplified DNA that was isolated from crosslinked chromatin bound to streptavidin beads and, therefore, were bound by NFATc1-bio-proteins. While no NFATc1 binding was detected in cells expressing BirA only, a similar binding was detected for NFATc1/A- and NFATc1/C-bio proteins (Fig. 6c). This shows that in WEHI B lymphoma cells NFATc1 can bind to the *Il10* gene *in vivo* and suppresses its transcription.

We had shown previously that in T cells NFATc1/C sumoylated at its C-terminal region is bound by histone deacetylases (HDACs) and suppresses the expression of the *Il2* gene^28^. To show whether in B cells NFATc1/αA is associated with HDACs we isolated chimaeric NFATc1/A-bio-protein from WEHI cells (Fig. 6e) and determined associated proteins in MS assays. Among the proteins bound to NFATc1/A-bio, in all sequencing reactions we detected—apart from CN peptides^29^ and IRF4 (ref. 30) as known NFATc binding partners—HDAC1, but no other HDAC nor any sirtuin member (Supplementary Table 1). This finding is supported by the increase of IL-10 RNA levels upon inhibition of HDAC activity in splenic B cells stimulated by IMQ and αIgM for 24 h, followed by co-incubation for 12 h with trichostatin A (TSA), a specific inhibitor of class I and II HDACs (ref. 31 and Fig. 6f). Moreover, treatment of splenic B cells with TPA and ionomycin (which mimics αIgM stimulation) led to an increase in binding of HDAC1 to the intronic site 2 of the *Il10* gene *in vivo.* The simultaneous increase of histone mark H3K4me3, a sign for gene transcription, at this site might reflect the transient nature of *Il10* expression under those stimulatory conditions (Fig. 6g).

## Discussion

Among the numerous mouse models used for studying human psoriasis^32^, the repetitive epicutaneous application of IMQ-containing Aldara cream to the shaved mouse skin represents a versatile and efficient experimental regimen to study early events of human disease^10^,^33^. The results of our study confirm the data of an earlier report on the important role of IL-10 produced by B cells in Aldara-induced skin inflammation^3^ and extend these observations by demonstrating the suppressive effect of NFATc1 on IL-10 production by B cells and on Aldara-mediated skin inflammation.

In mice, CD5^+^CD1d^hi^ B cells have been described as regulatory B cells (Bregs) that control experimental skin inflammation through the secretion of IL-10 (ref. 3). Similar to Tregs, Bregs tune immune reactions through affecting the release of cytokines^34^. They exert a regulatory influence on monocytes, T cells (on conventional CD4^+^T, Th1 and Th17 cells) and other B cells^35^. However, it is currently unclear and a matter of dispute whether similar to regulatory T (Treg) cells expressing Foxp3, Breg cells correspond to a particular subset of B cells with a specific developmental fate and function, or whether any B cell can be converted to IL-10-producing Breg/B10 cells by inflammatory signals^36^. Those signals play a major role in the differentiation to B10 cells that show, otherwise, often features of plasmablasts or plasma cells^37^.

While our study deals with the role of NFATc1 in controlling IL-10 production and skin inflammation by B cells, it is likely that—in addition to B cells—the enhanced expression of NFATc1 in other cells of psoriatic skin contributes to the induction and/or maintenance of disease. In T cells, NFATc1 stimulates the expression of several genes that are highly expressed during psoriasis, as the *Ccl3* and *Ccl4* chemokine genes. Several S100 Ca^++^-binding proteins that are strongly expressed in psoriatic skin^38^,^39^ are NFAT targets, or controlled by the Ca^++^/CN network. NF-κB is constitutively activated in psoriatic epidermis, and we showed recently that NF-κB factors support the induction of NFATc1/αA in murine lymphocytes^16^. Therefore, one may speculate that this event, that is, the NF-κB-mediated support of NFATc1/α induction, plays also a role in the development of disease. In KCs of human skin, both NFATc1 and c2 are expressed^40^,^41^,^42^, and NFATc1 was shown to exert a strong stimulatory effect on the proliferation of KCs. This is exemplified by RNAi-mediated knockdown of NFATc1 in an organotypic skin equivalent model leading to reduced epidermal thickness^41^.

The epicutaneous application of IMQ-containing Aldara cream to the murine skin does not only induce pathways downstream of TLR7 but also numerous other signalling events that induce inflammatory events. One of the first events following Aldara/IMQ application is the massive induction of epidermal cell death^25^, and apoptotic cells were shown to induce IL-10 in a large proportion of splenic B cells^43^. However, necroptosis seems also to play an important role in the death of KCs, and it is likely that by the release of numerous damage-associated molecular patterns, necroptotic KCs are potent triggers of inflammation. By their disintegration, necroptotic cells release many damage-associated molecular patterns, such as cytokines of the IL-1 family, the S100 proteins S100A8 and A9, nucleic acids, nucleoproteins, histones and heat-shock proteins^44^,^45^ that are able to induce inflammation. Although so far, the role of necroptosis in psoriasis has not been investigated^45^, the results of several murine models of the disease suggest necroptosis as a potent trigger of inflammation^46^,^47^,^48^.

In Aldara-induced skin inflammation, the massive death of KCs appears to induce the activation and differentiation of the murine immune system, including splenic B cells. Within 1–3 days of emollient application we observed the induction of NFATc1/αA in splenic B cells (Fig. 4b). However, application of Aldara cream for 3 days and longer did not lead to a further increase but to a decrease in NFATc1 levels. Persistent Aldara application led to a switch in IgM proteins and Ig heavy chains, and the differentiation of splenic B cells to Ab-producing cells (Fig. 4a and Supplementary Fig. 5). Therefore, with the onset of massive and persistent inflammation of KCs *in vivo*, splenic B cells become unresponsive to IgM signals, as it is documented in the inability to induce NFATc1/αA by α-IgM (Fig. 4b and Supplementary Fig. 7B,C). Instead, signals other than those mediated by α-IgM control the fate of such plasmablast-like B cells which, however, have the capacity to induce NFATc1/αA. This is reflected by the ability of splenic B cells from Aldara-treated mice to induce NFATc1/αA upon TPA+ionomycin treatment (Supplementary Fig. 7D).

The interplay between NFATc1 activity and IL-10 production is not only restricted to the generation of psoriasis-like symptoms but also involved in other autoimmune diseases. For CD4^+^ T cells from pediatric lupus patients high NFATc1 levels were detected that are correlated with an increased and prolonged CD154/CD40 ligand expression and glomerulonephritis^49^. Similar data were reported for the CD4^+^ T cells of MRL/*lpr* mice^50^, a murine model of human systemic lupus erythematosus. The increased CD154 expression on CD4^+^ T cells affects both B-cell differentiation^51^ and supports strongly the induction of NFATc1 in B cells^16^ that might result in repression of IL-10 production, which is characteristic for B cells of systemic lupus erythematosus patients^52^. Inactivation of NFATc1 in B cells led to an increase in IL-10 production and amelioration of symptoms of MOG-mediated experimental autoimmune encephalomyelitis^21^, and inactivating both *Nfatc1* and *Nfatc2* genes in T cells abolished the generation of experimental autoimmune encephalomyelitis symptoms^53^.

These and further data suggest that the NFATc1-mediated inhibition of IL-10 expression might be of general impact for the development of autoimmune diseases and a novel target how to treat them. Although the CN and NFAT inhibitors CsA and FK506 have been used for many years to treat autoimmune diseases, including psoriasis, their numerous side effects restrict their prolonged application. Instead, the selective inhibition of NFATc1 induction, in particular of NFATc1/αA, might specifically affect the (hyper-) activity of lymphoid cells. Our data presented here suggest that such a therapy could lead to an increase in IL-10 levels that, as shown in earlier studies, is of benefit for psoriasis patients^54^,^55^. Albeit there is currently no IL-10 therapy that passed clinical phase III studies^55^, enhanced IL-10 levels certainly support other therapies, such by antibodies raised against IL-17, IL-17 receptor and IL-23 that showed promising therapeutic results in phase III studies with psoriasis patients^56^. It remains a challenging task to elucidate whether the manipulation of NFATc1 and IL-10 activities, alone or together with those of IL-17 and IL-23, will be of benefit for patients suffering from psoriasis and other autoimmune diseases.

## Methods

### Mice and the induction of skin inflammation by Aldara

If not stated otherwise, 8–12-week-old C57BL/6 mice were used. Animal experiments were performed according to project licenses (No. 55.2–2523.01/10B and 32/14), which were approved and controlled by the ‘Regierung von Unterfranken, Würzburg'. *Nfatc1*^*f/f*^ *x mb1-cre* mice (C57BL/6) were described previously^21^. To generate mice bearing IL-10-deficient B cells, *Il10*^*f/f*^ mice^57^, were crossed with *mb1-cre* mice. To get mice double-deficient for NFATc1 and IL-10 (on C57BL/6 background), *mb1-cre* mice were crossed with mice bearing *Nfatc1*^*f/f*^ and *Il10*^*f/f*^ alleles. For Aldara-induced skin inflammation, mice were anaesthetized and their upper back was shaved. An amount of 62.5 mg Aldara cream (containing 5% IMQ, 25% isostearic acid, 2% benzyl alcohol, 2.2% cetyl alcohol, 3.1% stearyl alcohol, 3% white petrolatum, 3.4% polysorbate 60, 0.6% sorbitan monostearate, 2% glycerol, 0.2% methyl paraben, 0.02% propyl paraben, 0.5% xanthan gum in 52.98% water) were applied daily for 7 d. Control mice were treated with a comparable emollient cream (DAC Basiscreme, containing glycerol monostearate, 4%; cetyl alcohol, 6%; triglycerides, 7.5%; white petrolatum, 25.5%; macrogol-20-glycerol monostearate, 7%; propylenglycol, 10%; purified water, 40%). To score the severity of skin inflammation we used a mPASI, as described in detail previously^3^.

### Histology

Three micrometre paraffin sections of lesional skin were dewaxed and rehydrated with xylene and graded alcohols followed by heat-induced antigen retrieval by boiling in citrate buffer pH 6.0 (Dako) for 10 min. After quenching endogenous peroxidase with hydrogen peroxide, sections were incubated with the relevant Abs at 1 μg ml^−1^ (K6, K10, K14, Covance, New Jersey; S100A8, LifeSpan BioSciences, Seattle) or control Ab for 1 h at 37 °C. This was followed by incubation with the appropriate biotin-conjugated secondary Ab (VectorLabs, CA) and streptavidin-conjugated horseradish-peroxidase (VectorLabs) at room temperature for 1 h. Incubation with the peroxidase-specific substrate 3-amino-9-ethylcarbazole (Sigma-Aldrich) was used for visualization, with haematoxylin counterstaining. Abs against keratin 6 (K6, #PRB-169P; 2 μg ml^−1^), 10 (K10, #PRB-159P; 1 μg ml^−1^) and 14 (K14, #PRB-155P) were from Covance, against S100A8 (LS-B8014) from LifeSpan Biosciences, against B220 (#103202) and Gr-1 (#108402) from BioLegend, and against CD3G (#ab134096) from Abcam.

### B- and T-cell isolation and culture

Splenic B and CD4^+^T cells were isolated using Miltenyi's cell isolation kits (mouse; no. 130-090-862 and 130-049-201, respectively) to a purity of 95–98% as determined by flow cytometry. B cells were cultured in X-vivo 15 medium (Lonza)^21^. If not stated otherwise, splenic B cells were stimulated with 10 μg ml^−1^ α-IgM (F(ab')_2_ fragment goat anti-mouse IgM (Jackson ImmunoResearch Laboratories), or 10 μg ml^−1^ LPS (Sigma-Aldrich) and/or 5 μg ml^−1^ α-CD40 (R&D Systems), or with 100 ng ml^−1^ TPA and 0.5 μM ionomycin, or 1–10 μg ml^−1^ IMQ (MCE, MedChem Express) for 2–48 h, as indicated in the figures. Isolated primary T cells were incubated in X-vivo medium on wells pre-coated with α-CD3/CD28 (10 and 3 μg ml^−1^, respectively) for 72 h followed by intracellular staining and flow cytometry. Murine WEHI 231 cells were maintained in RPMI-1640 containing 10% FCS at 37 °C in 5% CO_2_.

### Expression of NFATc1-bio-proteins in WEHI 231 B cells

Full-length murine NFATc1/αA (gi:255759918 in NCBI database) and NFATc1/βC cDNAs (gi: 255759924) were amplified, fused to a bio/avidin-tag (ref. 58) and ligated into the retroviral expression vector pEGZ (ref. 59). The retroviral pMSCV-F-BirA vector was purchased from BCCM/LMBP (Gent-Zwijnaarde, Belgium). Retroviral particles were obtained after transfection of retroviral vectors, along with the retroviral packaging plasmids pHIT60 and pHIT123, into HEK 293T cells. After infection, WEHI cells were kept under selective conditions (using zeocin or puromycin) for 14 days. Positive integration and expression of NFATc1/αA-bio, NFATc1/βC-bio and/or BirA constructs was determined by intracellular streptavidin-fluorophore labelling and flow cytometry.

### Protein digestion and quantitative proteomic analysis

NFATc1-interacting proteins were eluted from streptavidin beads using a buffer containing 7 M urea, 2 M thiourea, 2% CHAPS, 10 mM biotin and digested with sequencing-grade trypsin (Trypsin Gold, Promega) using a modified FASP protocol^60^. After FASP digest, resulting tryptic peptides were concentrated to 20 μl by lyophilization. Five microlitre of 100 fmol μl^−1^ MassPrep Enolase Digestion Standard (Waters) were added to each sample and transferred into an autosampler vial. For nanoUPLC-MS analysis, 0.4 μl were used per injection. Samples were analysed in three technical replicates. Tryptic peptides were separated by reversed-phase nanoUPLC in direct injection mode on a Waters nanoAcquity System equipped with a C18 HSS-T3 75 μm × 250 mm column using a gradient from 4 to 40% B over 90 min as described before^61^. Buffer A was 0.1% formic acid in water+3% DMSO. Buffer B was 0.1% formic acid in acetonitrile +3% DMSO. The column was coupled to a nanoelectrospray source on a Waters Synapt G2-S mass spectrometer operated in ion-mobility enhanced, data-independent acquisition mode as described previously^61^. Resulting raw data files were processed by Protein Lynx Global Server (PLGS, v3.0.2) and database search was performed against the mouse UniProt Reference Proteome database supplemented with common contaminants (trypsin, bovine serum albumin, human keratins and so on) as described^61^. Data post processing and TOP3-based label-free quantification were performed in the ISOQuant Software^61^.

### Flow cytometry

B cells were washed once in cold PBS containing 0.1% BSA (FACS buffer) before blocking with anti-FcγRII/FcγRIII (2.4G2, BD Pharmingen, San Diego, CA). Stainings were performed on ice using conjugated mAbs (eBioscience, San Diego, CA, if not stated otherwise), diluted 1:300 in FACS buffer for surface marker and 1:200 for intracellular cytokine staining followed by incubation for 20 min. After washing with FACS buffer, cells were analysed on a FACS Canto II (BD) using FlowJo software (Tree star, Ashland, OR). The following Abs were used: B220-FITC (#11-0452-86), CD5-PE-Cy7 (#25-0051-81), CD1d-PE (#12-0011-81), CD138-APC (#142506, Biolegend), IgM-FITC (#11-5890-85), IgG1-PE (#12-4015-82), CD4-FITC (#11-0041-82), IL-2-APC (#17-7021-81), IFNγ-APC (#17-7311-82) and IL-10-PerCP (#45-7101-80). Abs against TNF−α−PE (#130-092-245) and IL-17-PE (#130-094-296) were from Miltenyi Biotec. For intracellular staining, the fixation and permeabilization kit (Plus Brefeldin A; eBioscience, Cat. no. 88-8823-88) was used according to manufacturer's recommendation.

### Western blotting

Whole-protein extracts from B cells were prepared by lysis of frozen cells in 60–100 μl RIPA buffer (pH 7.5, 50 mM Tris containing 150 mM NaCl, 1% Triton-X100 on ice for 20 min), followed by centrifugation and measurement of protein content. Western blots were performed by fractionating protein extracts (5–100 μg protein per lane) on SDS–polyacrylamide gel gels followed by immunodetection of NFATc1 using the 7A6 mAb (Santa Cruz or BD) or a polyclonal Ab (IG-457; ImmunoGlobe) raised against NFATc1 α-peptide. Protein loading was controlled by Ponceau red staining of membranes. Signals were visualized by chemoluminescence using Super Signal (Thermo Fisher Scientific). Images have been cropped for presentation. The uncropped data for blot and gel images can be found in Supplementary Figs 9–12.

### ChIP

ChIP assays were performed as described^62^,^63^ with slight modifications. In brief, 5 × 10^7^ WEHI-231 or primary splenic B cells were fixed, the reaction was quenched and washed cells were resuspended in 1 ml swelling buffer on ice for 30 min. Upon adding 40 μl of 10% NP-40, cells were passed 8 × through 21G needles, nuclei were collected and resuspended in 0.5 ml sonication buffer. Chromatin was sheared for 10 min (30 s pulses; 35% amplitude) on ice using an ultrasonic-disintegrator (Sonicor). DNA extracts of supernatants were checked for fragment sizes and quantified using a NanoDrop device (ThermoScientific). Chromatin was pre-cleared with 3 μg unrelated Ig Ab (CellSignaling, #2729p) and 40 μl immobilized protein G beads (ImmunoPure PIERCE, 50% slurry saturated with salmon sperm DNA, #16–157, Upstate; and 2% gelatin from cold water fish skin (FGEL), Sigma-Aldrich, #G7765). Chromatin in 300 μl sonication buffer was incubated with 25 μl of streptavidin agarose resin (ThermoScientific, #20347; 50% slurry saturated with Salmon Sperm DNA and FGEL) (Fig. 6c), or with 3 μg Ab against HDAC1 (Abcam,#ab109411) or H3K4me3 (Abcam, #ab8580)(Fig. 6g) at 4 °C overnight. Beads were carefully washed, and chromatin complexes were eluted twice by incubation with 250 μl elution buffer. Eluates were supplemented with 21 μl 5 M NaCl and 2 μl RNase (10 mg ml^−1^) for removal of crosslinks. DNA was extracted for PCR assays using the following primers from intron 4 of murine *Il10* gene:

*Il10*-forward: 5′- CACTAAGTTCCATAAACCGGAAA -3′; *Il10*-reverse: 5′- TGTGTAAAAGCCCCAGAACC -3′.

### RNA-Seq assays

RNA of deep-frozen splenic B cells or WEHI 231 B cells was extracted using Qiagen's RNeasy kit and Illumina's RNA purification beads. RNA-Seq libraries for next-generation sequencing were prepared from 600 ng starting material using Illumina's TruSeq RNA Sample Prep Kit V2 following the manufacturer's instruction. The resulting barcoded cDNA libraries were sequenced in one lane on an Illumina HiSeq 2000 (splenic B cells) or HiSeq 2500 (WEHI 231 B cells) platform for 50 nucleotides (single end). The data were loaded into ArrayExpress (accession no. E-MTAB-4665).

### Statistical analysis

Statistical analyses were performed using GraphPad (Prism) software, version 6.0. Data presented as mean and error bars in the figures represent±s.e.m. Unpaired *t*-tests were performed to evaluate the statistical significance of the data set. Statistical significances were calculated and indicated (***P<0.001, **P<0.005 and *P<0.05).

### Data availability

Data generated during this study is available in a public repository-ArrayExpress with accession no. E-MTAB-4665.

## Additional information

**How to cite this article:** Alrefai, H. *et al*. NFATc1 supports imiquimod-induced skin inflammation by suppressing IL-10 synthesis in B cells. *Nat. Commun.* 7:11724 doi: 10.1038/ncomms11724 (2016).

## Supplementary Material

Supplementary Information Supplementary Figures 1 - 12, Supplementary Table and Supplementary Reference
