# Corrigendum: NFATc1 supports imiquimod-induced skin inflammation by suppressing IL-10 synthesis in B cells

**DOI:** 10.1038/ncomms12421

**Published:** 2016-08-02

**Authors:** Hani Alrefai, Khalid Muhammad, Ronald Rudolf, Duong Anh Thuy Pham, Stefan Klein-Hessling, Amiya K. Patra, Andris Avots, Valesca Bukur, Ugur Sahin, Stefan Tenzer, Matthias Goebeler, Andreas Kerstan, Edgar Serfling

Nature Communications 7 Article number: 11724 doi: 10.1038/ncomms11724(2016); Published: 05 25 2016; Updated: 08 02 201610.1038/ncomms11724.

The affiliation details for Amiya K. Patra are incomplete in this Article. The correct affiliations are given below:

Institute of Translational and Stratified Medicine, Peninsula Schools of Medicine and Dentistry, University of Plymouth, Plymouth Science Park, Research Way, Plymouth PL6 8BU, UK.

Department of Molecular Pathology, Institute of Pathology, Julius-Maximilians-University, D-97080 Würzburg, Germany.

