# Supplementary material for: NFATc1 supports imiquimod-induced skin inflammation by suppressing IL-10 synthesis in B cells
Source: Nat Commun. 2016 May 25;7:11724. doi: 10.1038/ncomms11724 (PMC4894959; doi:10.1038/ncomms11724)
Supplement: Supplementary Information — Supplementary Figures 1 - 12, Supplementary Table and Supplementary Reference [file ncomms11724-s1.pdf]

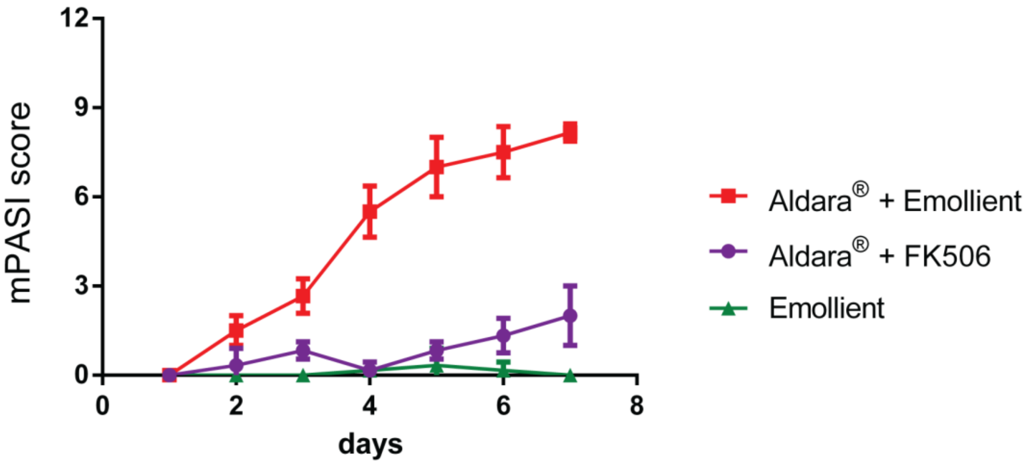

Supplementary Figure 1. FK506 suppresses Aldara®-induced skin inflammation. WT mice were shaved at the upper back and treated epicutaneously for 7 d with emollient cream alone, with Aldara® and emollient cream, or with Aldara® and Protopic® cream (Astellas; containing 0.1% FK506). In each of 3 assays, 3 mice were treated.

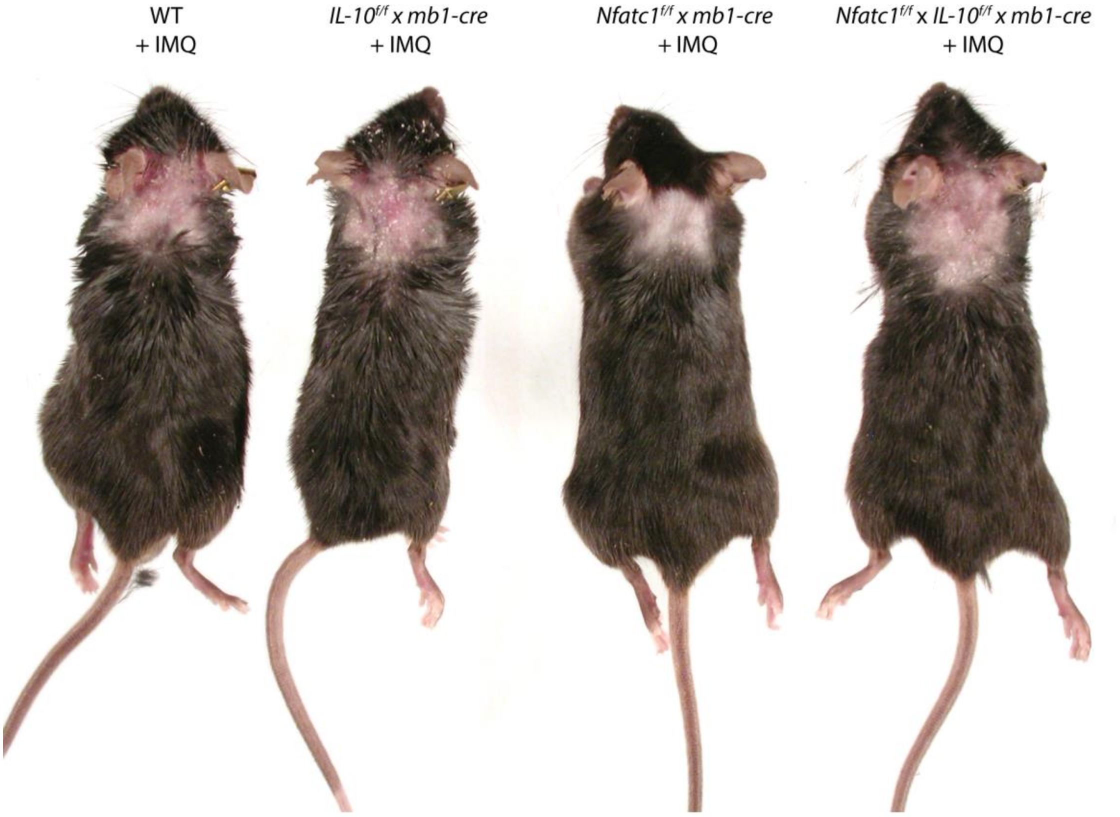

Supplementary Figure 2. IL-10 ablation abrogates the effect of NFATc1 inactivation on Aldara®-induced skin inflammation. WT mice or mice bearing IL-10-deficient (*Il10$^{f/f}$ x mb1-cre*), NFATc1-deficient (*Nfatc1$^{f/f}$ x mb1-cre*) or IL-10- and NFATc1-deficient B cells (*Nfatc1$^{f/f}$ x Il10$^{f/f}$ x mb1-cre*) were shaved at the upper back and treated epicutaneously with Aldara® cream for 7 d.

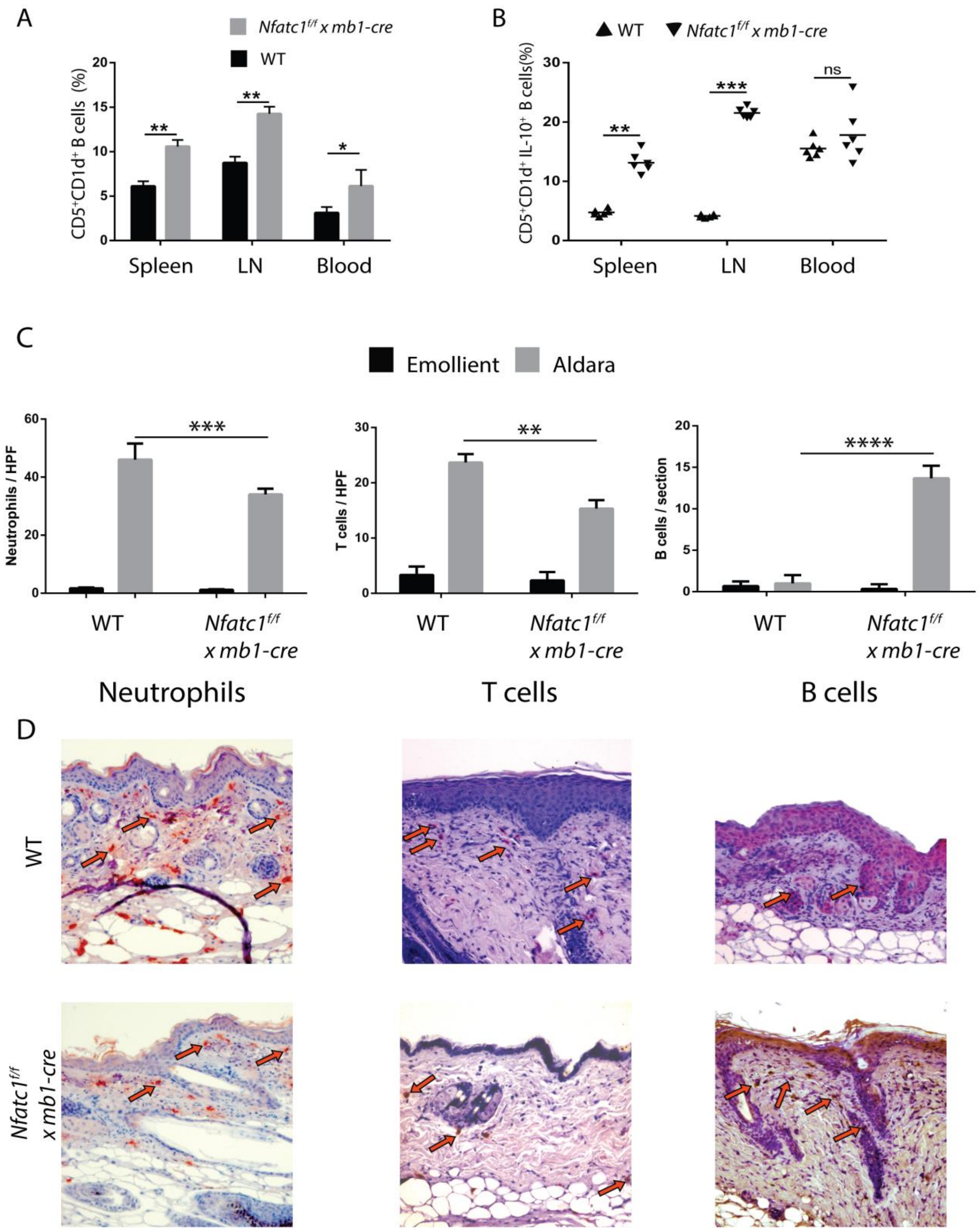

Supplementary Figure 3. Aldara® treatment leads to an increase in the number of B10 cells in spleen, lymph nodes (LN) and blood (A+B), and of infiltration of B cells into the skin (C+D). In (A) the proportion of $CD5^{+}CD1d^{+}$ B cells in preparations of

splenic, LN and blood B cells from WT and NFATc1-deficient mice is shown. In (B) the expression of IL-10 in $CD5^+CD1d^+$ B cells is presented. (C+D) Paraffin embedded sections of skin from WT and *Nfatc1$^{f/f}$ x mb1-cre* mice treated either with emollient or with Aldara® were stained with H&E or immunolabeled with Abs raised against Gr-1 (for neutrophils/granulocytes), CD3 (T cells) or B220 (B cells). Arrows indicate cells stained by Abs. In each set of experiments, skin sections of three mice were prepared, and – for neutrophils and T cells - the cells in 10 high power fields (HPF) were counted. For B cells, cells from whole sections were counted. For A-C, two-tailed unpaired Student's t-test was used. Data are shown as means ± SEM.

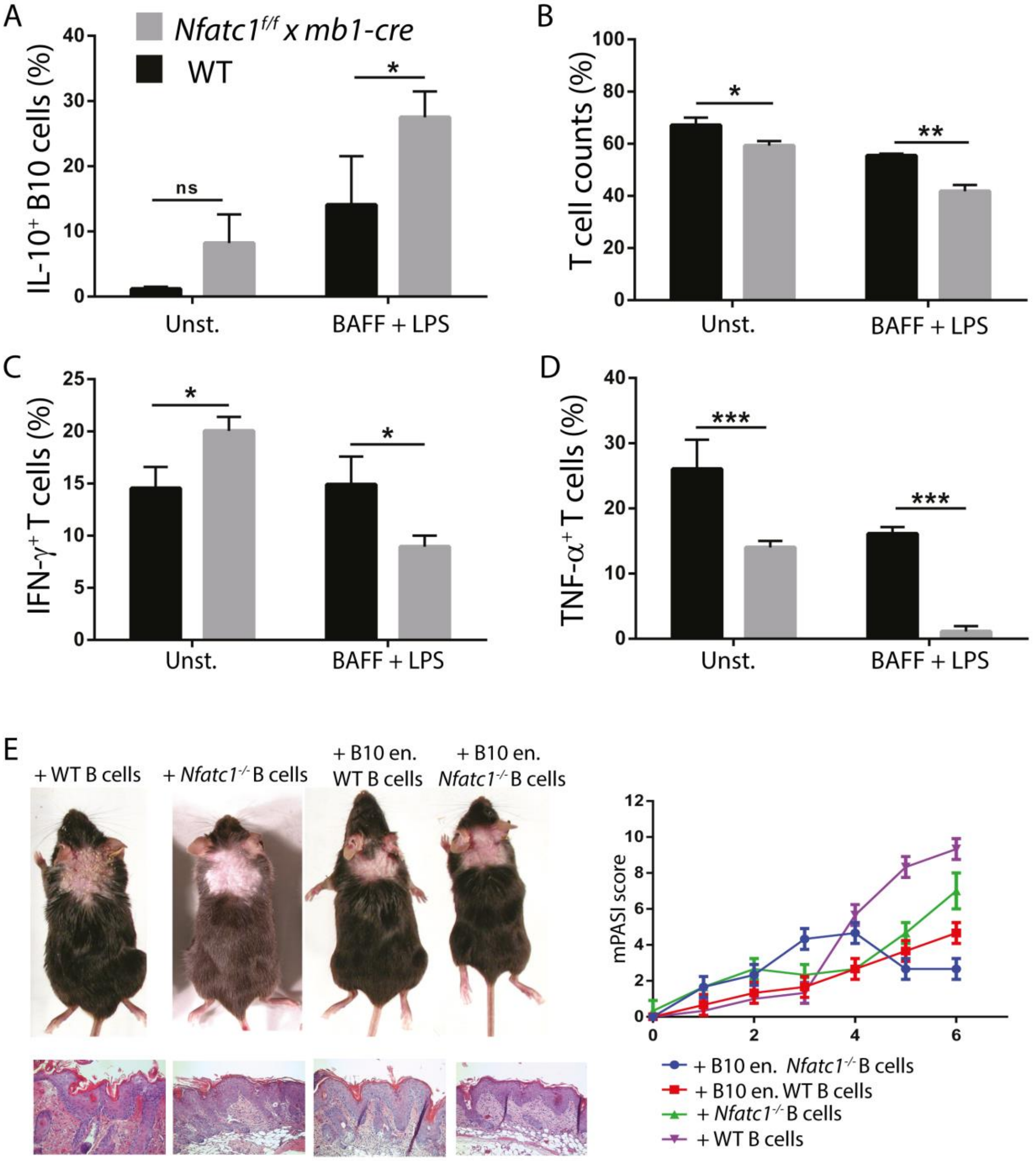

Supplementary Figure 4 Enriched B10 cells suppress Aldara®-mediated skin inflammation as well as proliferation and inflammatory cytokine expression of T cells. (A) Enrichment of IL-10-producing B cells upon treatment with LPS and BAFF for 3 d. (B-D) Effect of enriched B10 cells on T cell proliferation (B), on the production of IFN-γ (C) and TNF-α (D) by $CD4^+$T cells. $5x10^6$ B cells containing enriched B10 cells (as

prepared in [A]) were injected intraperitoneally into mice. 48 h later, the T cells of those mice were analysed. (E) Adoptive transfer of enriched B10 cells ameliorates skin inflammation by Aldara®. $5x10^6$ freshly prepared splenic B cells or enriched B10 cells were adoptively transferred into WT mice, and their mPASI was determined. Shown are mice and H & E-stained skin sections after topical Aldara®-application for 6 d. For A-D, two-tailed unpaired Student's t-test was used. Data are shown as means ± SEM.

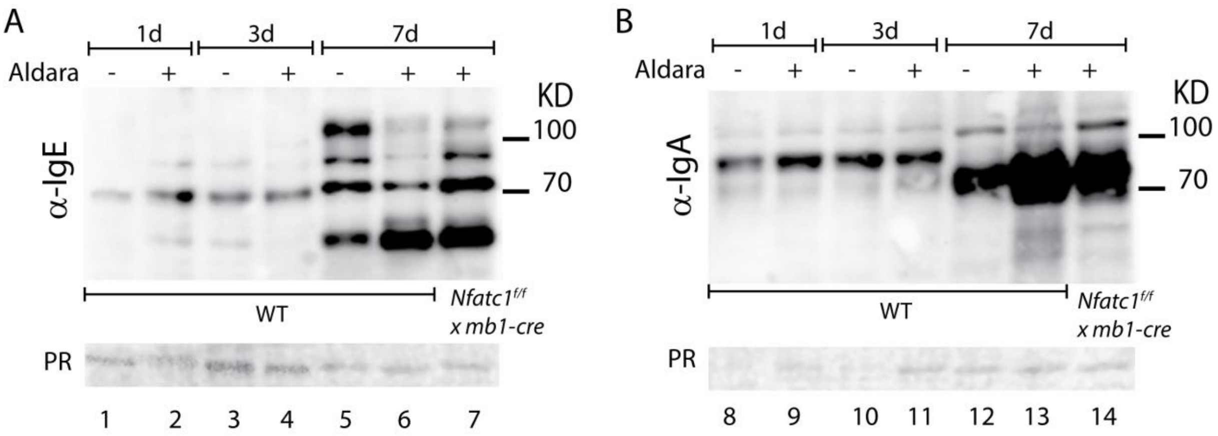

Supplementary Figure 5. Aldara® treatment leads to differentiation of splenic B cells to Ab-producing cells. In (A) and (B), mice were treated with Aldara® (+) or emollient cream (-) for 1, 3 or 7 d. Their splenic B cells were isolated, whole protein extracts prepared and immunoblotted with Abs raised against murine IgE (lanes 1-7) or IgA (8-14). PR, ponceau red.

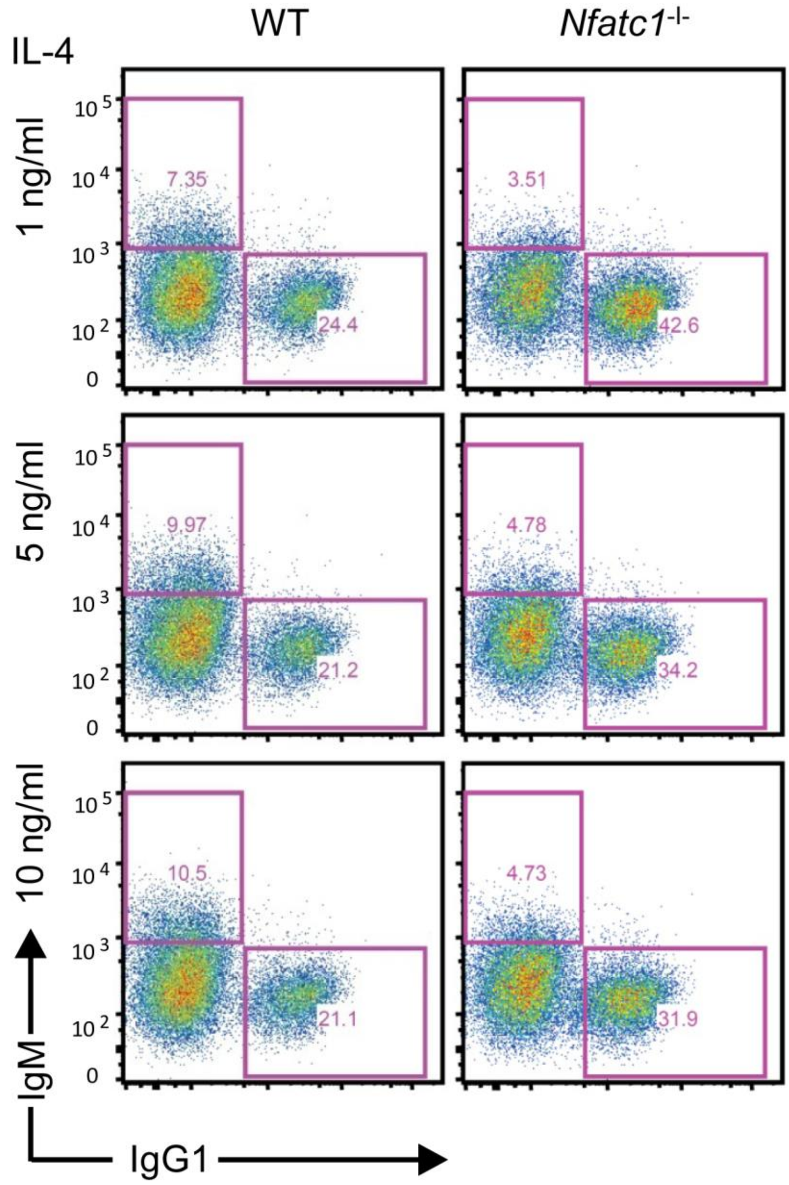

Supplementary Figure 6. NFATc1 ablation supports the differentiation of splenic B cells to IgG1-producing cells *in vitro.* Freshly prepared splenic B cells from WT and *Nfatc1*$^{fl/fl}$ *x mb1-cre* mice were cultured for 4 d on 3T3 feeder layer cells expressing CD40L and BAFF [1] in the presence of IL-4 as indicated. The expression of Ig proteins was determined by flow cytometry.

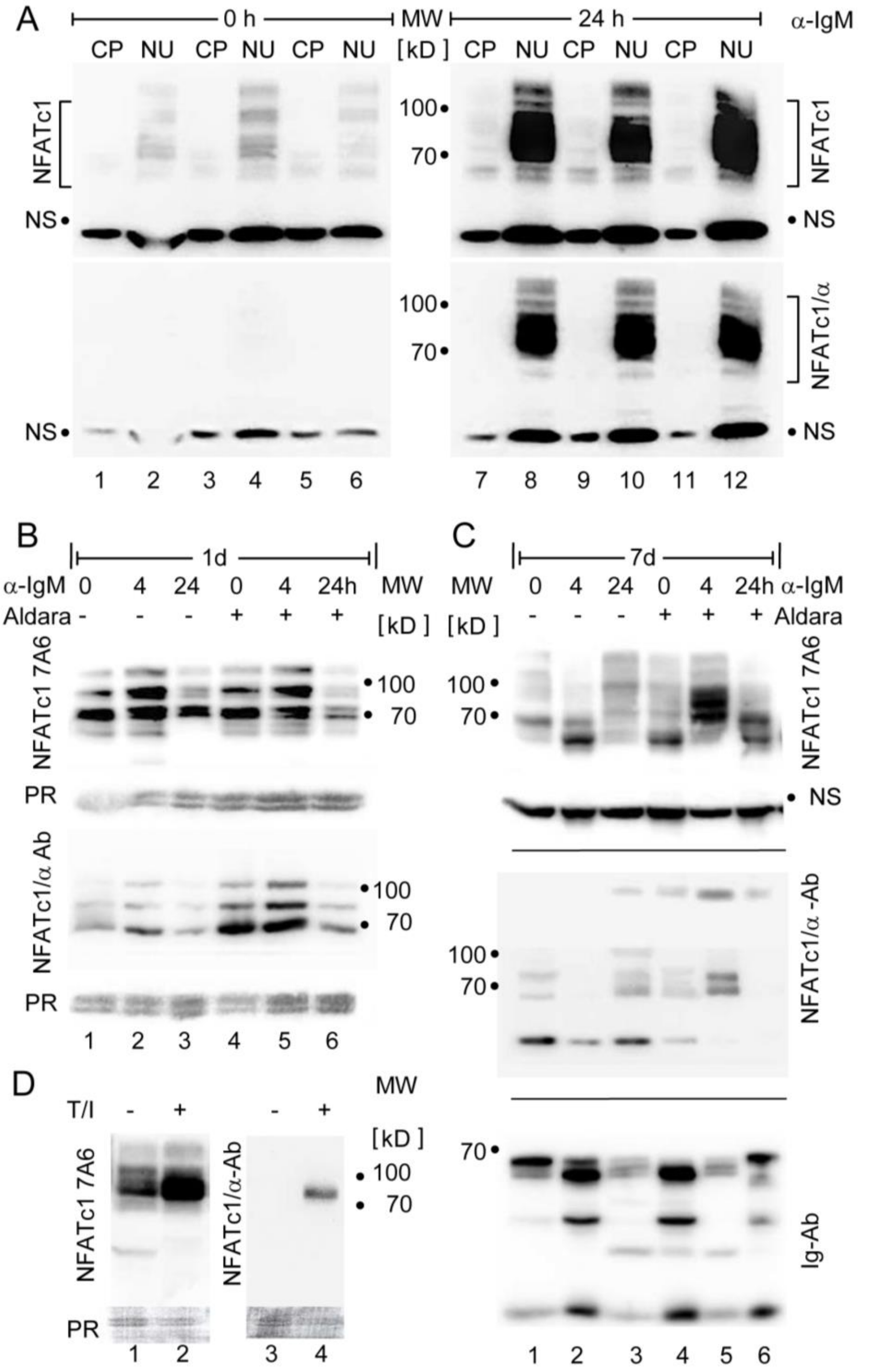

Supplementary Figure 7. Induction of nuclear NFATc1/α in splenic B cells and its suppression by Aldara® treatment of mice. (A) Splenic B cells derived after treatment with Aldara® from young WT mice (6 weeks of age: lanes 1+2 and 7+8), older mice (12 months of age: lanes 3+4 and 9+10) or very old mice (16 months of age: lanes

5+6 and 11+12) were left untreated (0 h), or were stimulated by 10 μg/ml αIgM for 24 h. Their cytoplasmic and nuclear proteins were isolated and immunoblotted with Abs against NFATc1 (top) or NFATc1/α (bottom). (B+C) Immunoblots of whole cell protein of splenic B cells obtained from mice treated with Aldara® (+) or emollient cream (-) for 1 or 7 d. Blots were probed with NFATc1 (7A6) mAb, the NFATc1α-peptide Ab or an Ab directed against murine Ig as indicated. NS, non-specific bands. (D) Immunoblots of whole cell protein of splenic B cells from mice treated with Aldara® for 7d. Cells were left untreated (-) or treated for 24 h by TPA and ionomycin (+, T/I).

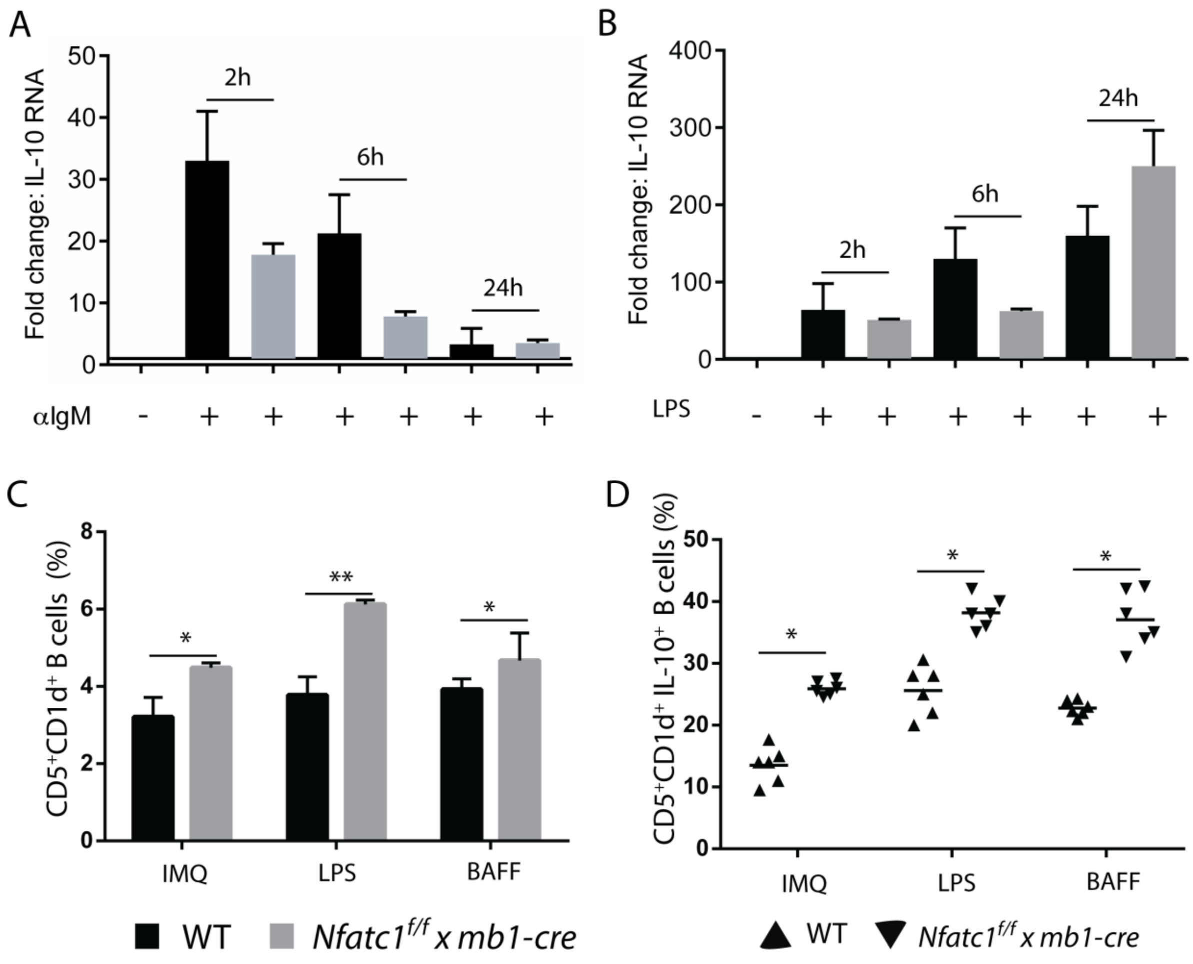

Supplementary Figure 8. Induction of IL-10 RNA in WT and NFATc1-deficient splenic B cells by 10 μg/ml αIgM (A) or LPS (B) for 2-24 h *in vitro*. IL-10 RNA was quantified by RT PCR. In (C) and (D), the effect of IMQ, LPS or BAFF treatment of splenic B cells for 48 h on the occurrence of $CD5^{+}CD1d^{+}$ B cells among splenic B cells (C) and the expression of IL-10 in $CD5^{+}CD1d^{+}$ B cells (D) was measured by flow cytometry. Data are shown as means ± SEM and two-tailed unpaired Student's t-test was used for statistical analysis.

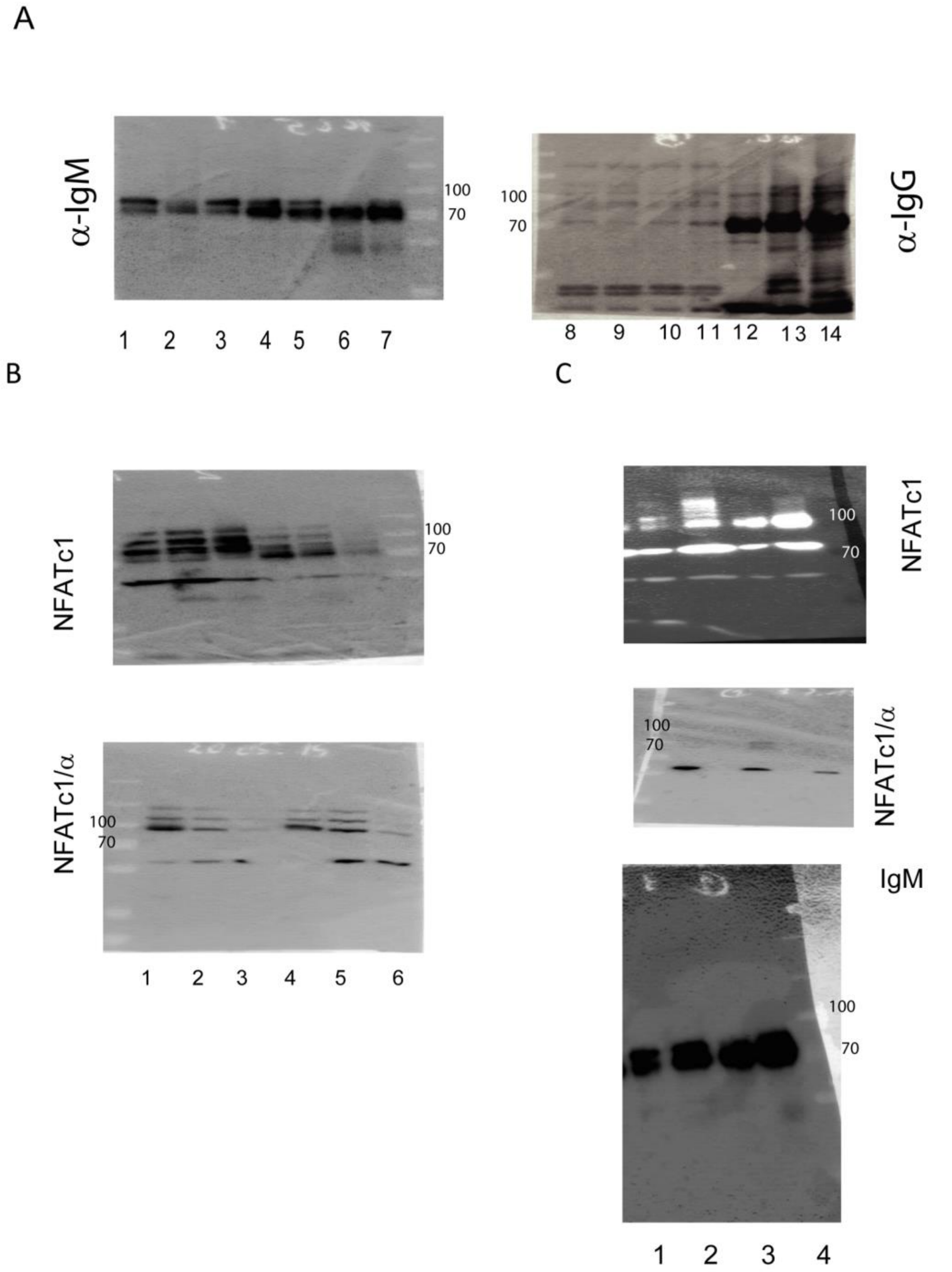

Supplementary Figure 9. Uncropped data for Figure 4.

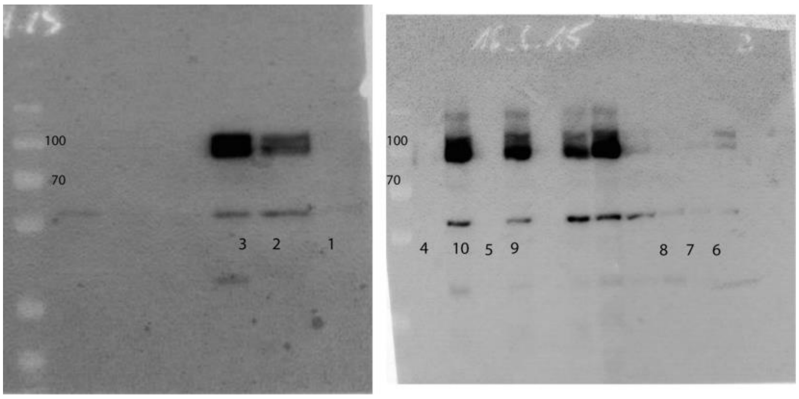

Supplementary Figure 10. Uncropped data for Figure 5C.

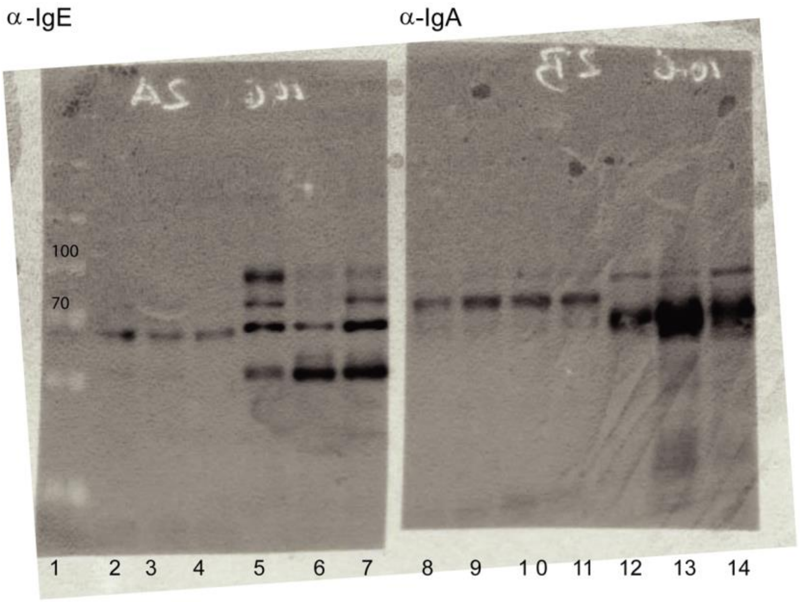

Supplementary Figure 11. Uncropped data for supplementary Figure 5.

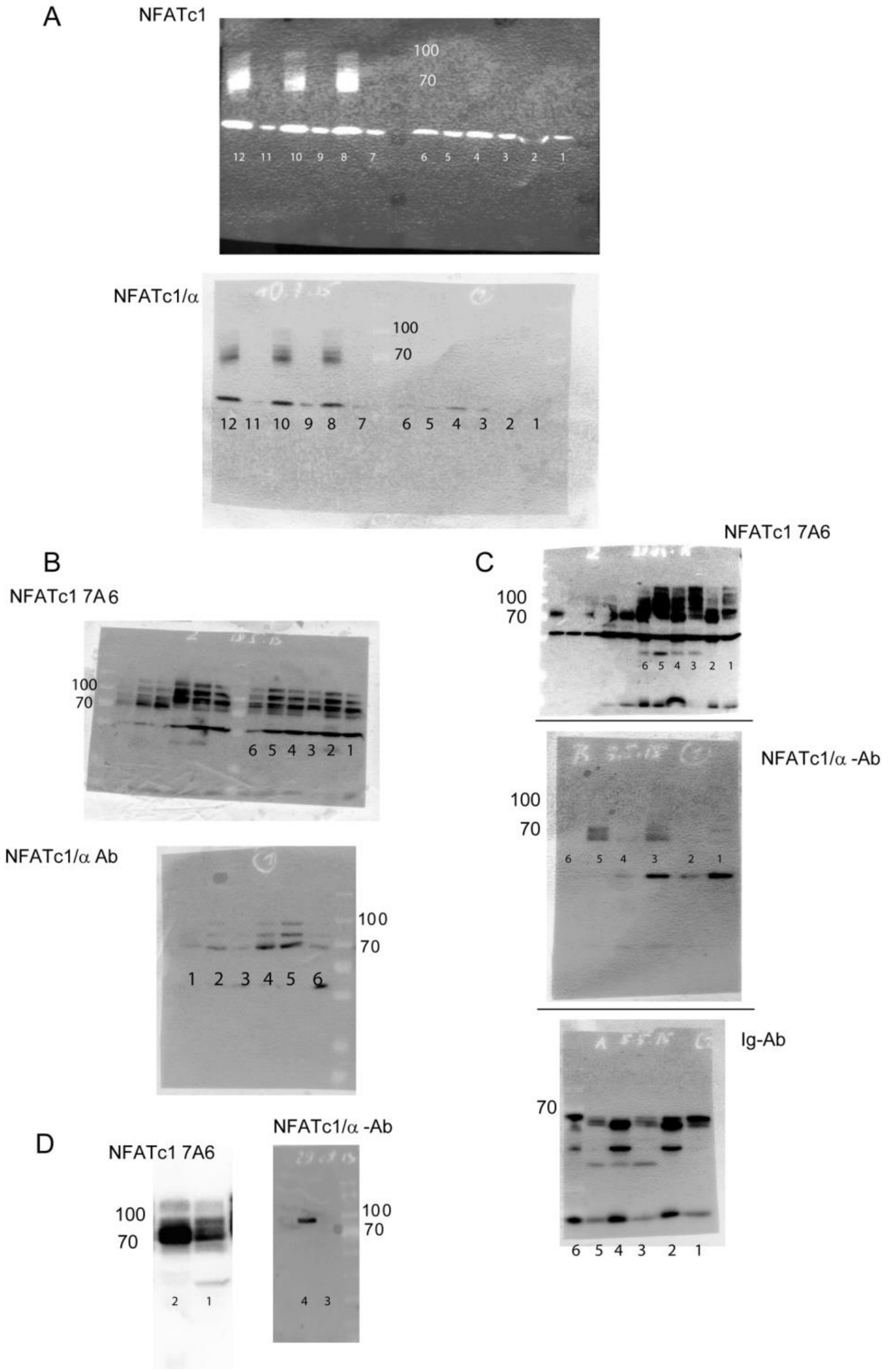

Supplementary Figure 12. Uncropped data for supplementary Figure 7.

Supplementary Table 1. Proteins associated with NFATc1/A-bio in WEHI 231 B cells. Shown are proteins that were sequenced in 6 independent MS assays.

UniProtKB-Nr.	Gene Symbol	Protein name	No. of unique peptides	Coverage
**Calcineurin Components/Phosphatases**				
P48453	*Ppp3cb*	Protein phosphatase 3, calcineurin, cat. subunit β	13	31,43
Q63810	*Ppp3r1*	Protein phosphatase 3, calcineurin, reg. subunit 1	3	22,94
Q6P1F6	*Ppp2r2a*	Protein phosphatase 2, regulatory subunit B, α	6	13,87
**Protein Kinases**				
P11440	*Cdk1*	Cyclin-dependent kinase 1	3	16,16
Q07832	*Plk1*	Polo-like kinase 1	10	21,89
Q9R0N0	*Galk1*	Galactokinase 1	5	13,78
Q9D7G0	*Prps1*	Phosphoribosyl pyrophosphate synthetase 1	8	38,68
P70218	*Map4k1*	Mitogen-activated protein kinase kinase kinase kinase 1	4	5,68
O70126	*Aurkb*	Aurora kinase B	9	31,88
**Cytoskeleton/Scaffold/Adapters**				
Q61033	*Tmpo I*	Thymopoietin	18	34,63
P61161	*Actr2*	Actin-related protein 2	5	26,14
Q99JY9	*Actr3*	Actin-related protein 3	8	25,6
Q922F4	*Tubb6*	Tubulin beta-6 class V	12	41,16
P61963	*Dcaf7*	DDB1 and CUL4 associated factor 7	2	8,19
P62259	*Ywhae*	Tyrosine 3-monooxygenase/tryptophan 5-monooxygenase activation protein, ε	7	37,65
Q9R0L6	*Pcm1*	Pericentriolar material 1 protein	23	17,23
Q80YR5	*Safb2*	Scaffold attachment factor B2	5	7,57
Q61897	*Krt33b*	Keratin 33b	8	29,7
Q9JIF0	*Prmt1*	Protein arginine N-methyltransferase 1	8	26,42

**Splicing/Polyadenylation**				
P29341	*Pabpc1*	Polyadenylate-binding protein 1	13	31,92
Q99LF4	*Rtcb*	RNA 2',3'-cyclic phosphate and 5'-OH ligase	7	16,63
Q9D883	*U2af1*	Splicing factor U2AF 35 kDa subunit	3	14,23
Q99KP6	*Prpf19*	Pre-mRNA-processing factor 19	5	12,5
Q9CX86	*Hnrnpa0*	Heterogeneous nuclear ribonucleoprotein A0	3	12,46
Q3U0V1	*Khsrp*	KH-type splicing regulatory protein	8	13,1
Q8R326	*Pspc1*	Paraspeckle protein 1	6	12,05
P84089	*Erh*	Enhancer of rudimentary homolog	2	42,31
**Ribosomal Proteins/Ribosome**				
P62908	*Rps3*	Ribosomal protein S3	11	62,14
P62281	*Rps11*	Ribosomal protein S11	8	62,03
P63323	*Rps12*	Ribosomal protein S12	3	20,45
P62245	*Rps15a*	Ribosomal protein S15a	4	23,08
P62267	*Rps23*	Ribosomal protein S23	3	19,58
P62855	*Rps26*	Ribosomal protein S26	2	20,87
P35979	*Rpl12*	Ribosomal protein L12	5	43,03
P84099	*Rpl19*	Ribosomal protein L19	2	11,73
Q8BP67	*Rpl24*	Ribosomal protein L24	4	33,12
P62889	*Rpl30*	Ribosomal protein L30	3	29,57
O35130	*Emg1*	EMG1 N1-specific pseudouridine methyltransferase	9	50,82
Q9EQ61	*Pes1*	Pescadillo homolog 1, containing BRCT domain	6	10,45
**Protein Synthesis**				
O70194	*Eif3d*	Eukaryotic translation initiation factor 3, subunit D	7	14,96
Q91VC3	*Eif4a3*	Eukaryotic translation initiation factor 4A3	13	34,31
Q9D8N0	*Eef1g*	Eukaryotic translation elongation factor 1 γ	12	24,94
Q9Z1Q9	*Vars*	Valyl-tRNA synthetase	26	25,18
**RNA Binding/Export**				
Q9CWZ3	*Rbm8a*	RNA binding motif protein 8a	2	15,52
Q8C570	*Rae1*	RAE1 RNA export 1 homolog	5	12,77

Q8R2U0	*Seh1l*	SEH1-like (S. cerevisiae)	2	12,5
Q91VR5	*Ddx1*	DEAD (Asp-Glu-Ala-Asp) box polypeptide 1	18	34,46
**Nuclear/Membrane Import/Export**				
P46061	*Rangap1*	RAN GTPase-activating protein 1	17	33,45
Q8BKC5	*Ipo5*	Importin 5	8	14,59
Q9JIH2	*Nup50*	Nucleoporin 50	11	33,69
P70168	*Kpnb1*	Karyopherin (importin) beta 1	13	19,63
Q6P5F9	*Xpo1*	Exportin 1	21	27,17
P61222	*Abce1*	ATP-binding cassette, sub-family E (OABP), member 1	12	25,04
**Transcription Factors**				
P56477	*Irf5*	Interferon regulatory factor 5	7	20,93
Q9WTK5	*Nfkb2*	NF-kB2, p52/p100	2	2,78
Q99K48	*Nono*	Non-POU domain-containing octamer-binding protein	5	11,42
Q921F2	*Tardbp*	TAR DNA binding protein	6	19,57
Q64287	*Irf4*	Interferon regulatory factor 4	10	29,56
Q8BFT2	*Haus4*	HAUS augmin-like complex, subunit 4	10	26,45
**Transcription/Chromatin**				
O09106	*Hdac1*	Histone deacetylase 1	5	9,54
Q62318	*Trim28*	Tripartite motif-containing 28	8	14,51
Q9JIX8	*Acin1*	Apoptotic chromatin condensation inducer 1	9	8,74
Q8BY71	*Hat1*	Histone aminotransferase 1	3	9,13
O35381	*Anp32a*	Acidic (leucine-rich) nuclear phosphoprotein 32, family member A	5	26,32
**Signalling**				
P63001	*Rac1*	RAS-related C3 botulinum substrate 1	4	19,79
Q05144	*Rac2*	RAS-related C3 botulinum substrate 2	3	15,62
P27870	*Vav1*	Vayv 1 oncogene	15	20,00
Q9CQM9	*Glrx3*	Glutaredoxin 3	6	29,08
P68040	*Rack1*	Receptor for activated C kinase 1	14	62,15
**Metabolism**				
P06151	*Ldha*	Lactate dehydrogenase A	10	30,72

Q61753	*Phgdh*	3-phosphoglycerate dehydrogenase	14	37,9
P12382	*Pfkl*	Phosphofructokinase, liver, B-type	13	23,08
Q9JLJ2	*Aldh9a1*	Aldehyde dehydrogenase 9 subfamily A1	2	7,09
Q3THK7	*Gmps*	Guanine monophosphate synthetase	9	15,73
**Ubiquitinylation/Sumoylation**				
P46935	*Nedd4*	Neural precursor cell expressed, developmentally down-regulated 4	16	23,90
Q8CFI0	*Nedd4l*	Neural precursor cell expressed, developmentally down-regulated gene 4-like	2	2,89
**DNA replication/Chromosome structure**				
P25206	*Mcm3*	Minichromosome maintenance deficient 3	13	18,72
P49717	*Mcm4*	Minichromosome maintenance deficient 4 homolog	10	14,27
P49718	*Mcm5*	Minichromosome maintenance deficient 5, cell division cycle 46	15	25,24
Q61881	*Mcm7*	Minichromosome maintenance deficient 7	11	21,70
Q8BG81	*Poldip3*	Polymerase delta-interacting protein 3	4	14,76
Q9CU62	*Smc1a*	Structural maintenance of chromosomes 1A	7	6,08
Q8BK67	*Rcc2*	Regulator of chromosome condensation 2	14	38,08
P17918	*Pcna*	Proliferating cell nuclear antigen	2	5,75
Q810D6	*Grwd1*	Glutamate-rich WD repeat-containing 1	3	8,74
**Heat shock/Stress factors/Chaperonines/Proteosome**				
Q9QYI3	*Dnajc7*	DnaJ heat shock protein family (Hsp40) member C7	7	19,84
Q61699	*Hsph1*	Heat shock 105kDa/110kDa protein 1	12	21,79
P11983	*Tcp1*	T-complex protein 1	12	33,81
P80318	*Cct3*	Chaperonin containing Tcp1, subunit 3 (gamma)	10	21,83
P80316	*Cct5*	Chaperonin containing Tcp1, subunit 5 (epsilon)	9	18,67
P80317	*Cct6a*	Chaperonin containing Tcp1, subunit 6a (zeta)	16	33,33
P80313	*Cct7*	Chaperonin containing Tcp1, subunit 7 (eta)	5	16,36
P42932	*Cct8*	Chaperonin containing Tcp1, subunit 8 (theta)	14	31,02
P62334	*Psmc6*	Proteasome (prosome, macropain) 26S subunit, ATPase, 6	10	30,08
**Miscellaneous**				
Q61584	*Fxr1*	Fragile X mental retardation gene 1, autosomal homolog	8	19,35

P70698	*Ctps1*	Cytidine 5'-triphosphate synthase 1	9	17,26
Q80SW1	*Ahcyl1*	S-adenosylhomocysteine hydrolase-like 1	21	39,62
P34884	*Mif*	Macrophage migration inhibitory factor	3	35,65
Q3V3R1	*Mthfd1l*	Methylenetetrahydrofolate dehydrogenase (NADP+ dependent) 1-like	9	11,16
Q64737	*Gart*	Phosphoribosylglycinamide formyltransferase	21	33,96
Q9D1M0	*Sec13*	SEC13 homolog, nuclear pore and COPII coat complex component	4	23,91
Q9R0Q7	*Ptges3*	Prostaglandin E synthase 3 (cytosolic)	4	38,12
Q9DCL9	*Paics*	Phosphoribosylaminoimidazole carboxylase, succinocarboxamide synthetase	12	32,94
O70325	*Gpx4*	Glutathione peroxidase 4	5	26,4
P17751	*Tpi1*	Triosephosphate isomerase 1	7	42,47
Q9CQE8	*C14orf166*	Chromosome 14 open reading frame 166	6	33,61
P50431	*Shmt1*	Serine hydroxymethyltransferase 1	8	27,2
Q91VI7	*Rnh1*	Ribonuclease/angiogenin inhibitor 1	12	40,35

References:

1. Nojima, T., *et al.* In-vitro derived germinal centre B cells differentially generate memory B or plasma cells in vivo. *Nat Commun* **2**, 465 (2011)
